# Molecular genetic associations between a prominent serotonin transporter gene polymorphism (5‐HTTLPR/rs25531) and individual differences in tendencies toward autistic traits and generalized internet use disorder in China and Germany

**DOI:** 10.1002/brb3.2747

**Published:** 2022-09-15

**Authors:** YingYing Zhang, Shuxia Yao, Helena Schmitt, Benjamin Becker, Keith M. Kendrick, Christian Montag

**Affiliations:** ^1^ Department of Molecular Psychology Institute of Psychology and Education, Ulm University Ulm Germany; ^2^ The Clinical Hospital of Chengdu Brain Science Institute, Ministry of Education Key Lab for Neuroinformation University of Electronic Science and Technology of China Chengdu China

**Keywords:** autistic traits, China, Germany, internet use disorder, serotonin transporter gene (5‐HTTLPR/rs25531)

## Abstract

**Background:**

The serotonin transporter polymorphism 5‐HTTLPR is an extensively investigated genetic marker of autistic traits or autism spectrum disorder, and recently has also been studied in the realm of internet use disorder (IUD), yet the findings remain controversial. Therefore, the present study aimed to explore associations between 5‐HTTLPR (also including SNP rs25531) and autistic traits/IUD tendencies and to assess whether the relationship between autistic traits and IUD tendencies varies by this genetic marker in participants from China and Germany.

**Methods:**

A total of 540 Chinese and 563 German subjects were genotyped for 5‐HTTLPR/rs25531 and completed the Adult Autism Spectrum Quotient questionnaire and the short version of the Internet Addiction Test.

**Results:**

Carriers of the low expressing S'S’ genotype (S, L_G_) showed significantly higher levels of autistic traits than the high expressing allele (e.g. L_A_) carriers in both samples. There was no significant effect of 5‐HTTLPR/rs25531 on IUD either in the Chinese or Germany samples, whereas positive correlations between autistic traits and IUD varied by 5‐HTTLPR/rs25531 genotypes and also differed between Chinese and German samples. In the Chinese sample, positive correlations were mainly driven by S'S’ and S'L' carriers, while they were mainly determined by S'L’ and L'L' carriers in the German sample. Further analyses revealed that the associations between autistic traits and IUD tended in parts to be more strongly pronounced in the complete German sample compared to the complete Chinese sample, and also varied depending on 5‐HTTLPR/rs25531 genotypes (in S'S’ carriers: China > Germany; in S'L’ and L'L’ carriers: China < Germany; both in terms of more positive associations).

**Conclusions:**

Our findings suggest carriers of low expressing alleles (S, L_G_) are more likely to show higher autistic traits in both Chinese and German samples. Furthermore, the present work shows that both 5‐HTTLPR/rs25531 and cultural differences might be of relevance to understand associations between autistic traits and IUD tendencies, but this needs to be further backed up.

## Graphical abstract

We investigated the role of the a prominent serotonin transporter gene polymorphism for autistic traits and internet use disorder. Molecular genetic associations with autistic traits could be observed, but no associations with Internet Use Disorders appeared.

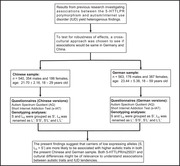

## 1 INTRODUCTION

Autistic traits measured in the general population follow a smooth distribution, with the most severe end of the continuum being associated with clinical recognition of autism spectrum disorder (ASD) (Baron‐Cohen et al., 2001; Lundström et al., 2012; Ronald et al., 2006). It has been well established in twin studies that genetics play a tremendous role to understand the risk for ASD, with heritability rates ranging from 64% to 91% (Folstein & Rutter, 1977; Tick et al., 2016). Correspondingly, a genome‐wide association study provided further evidence for a shared genetic etiology and biology between ASD and autistic traits, which suggests that genetic studies of autistic traits might yield novel ASD genes and loci (Bralten et al., 2018).

Among the candidate genetic factors for association studies in the etiology of ASD, the serotonin transporter (5‐HTT) gene (SLC6A4) is one of the most promising (Heils et al., 1996). The SLC6A4 gene bears a 44 base pair insertion/deletion polymorphism in the promoter region (5‐HTT gene linked polymorphic region, 5‐HTTLPR, (Heils et al., 1996)), which mediates the reuptake of serotonin from synaptic spaces into presynaptic neurons. 5‐HTTLPR was initially considered functionally bi‐allelic, with a short allele (S, 14 repeats) and a long allele (L, 16 repeats). Relative to the L allele, the S allele has been reported to result in lower transcription activity in mRNA levels, serotonin binding, and reuptake (Little et al., 1998). So far, research findings regarding associations between 5‐HTTLPR and ASD yielded highly heterogeneous findings, with some studies reporting the S allele to be the risk variant for ASD (e.g., Arieff et al., 2010a; Meguid et al., 2015), while other studies suggest the L allele as the risk variant (e.g., Cho et al., 2007), whereas recent reviews and meta‐analyses have reported no association (e.g., Nuñez‐Rios et al., 2020; Wang et al., 2019; Wei et al., 2021). One possible explanation for these inconsistent results observed in the literature might be the different grouping of the genotypes (SS, SL, and LL in S+/S‐ or L+/L‐ groups), or not taking into account an important single nucleotide polymorphism (SNP; rs25531(A 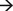 G)), which is known to further modulate the mRNA expression of the SLC6A4 gene (Hu et al., 2006). In detail, SNP rs25531 has been identified within the L allele, further dividing the L allele into L_A_ and L_G_ and thus leading to a tri‐allelic genotyping classification (S, L_A_, and L_G_) (Nakamura et al., 2000). The 5‐HTT protein transcription level of L_G_ is almost equivalent to that of S, with both being lower than L_A_. This approach reclassified L_G_ and S as S’ and L_A_ as L’ (Zalsman et al., 2006). Hence, if the rs25531 genotype is not taken into account, L_G_ may have been misclassified as a “high expression” variant in previous studies. Of note, to our knowledge, the tri‐allelic 5‐HTTLPR/rs25531 classification method has not been used for investigating the relationship between 5‐HTTLPR and autistic traits in the general population until now, so this will be one of the aims of the present study. This is not the only aim of the present study. It is also aimed to touch upon a less studied field in the context of autistic traits, namely its link with internet use disorder(s) (IUDs). The positive association between autistic traits and IUD was robustly presented earlier in the literature (Finkenauer et al., 2012; Romano et al., 2014; Zhang et al., 2021).

IUD is a condition describing addictive or problematic behaviors related to online activities such as gaming, gambling, shopping, and social communication (for a recent taxonomy see Montag et al., 2021). Factors disposing a person toward developing IUD are complex. The so‐called I‐PACE model suggests that a complex interaction of person–affect–cognition and execution variables might be at the heart of IUD (Brand et al., 2016). In the context of the present study, we consider that a critical factor may be individual genetic variation and it is of prominent interest that a critical element of the person‐variable of the I‐PACE model represents genetics (Brand et al., 2016). Several studies have investigated the molecular genetic basis of IUD (Montag et al., 2012; Paik et al., 2017; Sindermann et al., 2021), and some even studied links between 5‐HTTLPR and IUD (for instance, with a focus on internet gaming disorder, for an overview see Montag & Reuter (2017)). However, the findings on the relationship between 5‐HTTLPR and IUD were all based on a bi‐allelic (S and L) classification and were inconsistent so far: Lee et al. (2008) found that the homozygous S/S genotype was more prevalent among individuals with IUD (Lee et al., 2008), and Sun et al. (2016) indicated that individuals with S/S genotype showed higher levels of IUD (in particular in male participants, but the sample was very small). Nonetheless, a more recent work failed to detect a significant relationship between IUD and 5‐HTTLPR (Cerniglia et al., 2020). Hence, it is necessary to assess the relationship between 5‐HTTLPR and IUD by also taking into account the SNP rs25531.

Cultural differences, as a common environmental factor interacting with genetics, might be a further potential factor influencing the relationships between 5‐HTTLPR and IUD/autistic traits. The present work therefore investigated samples from China and Germany since both prevalence of 5‐HTTLPR/rs25531 genotypes and IUD in populations vary by ethnic groups. For 5‐HTTLPR/rs25531, the frequencies of S'S’, S'L’, and L'L’ variants in Han‐Chinese subjects (Ho et al., 2016) were different from those of Caucasians (Zalsman et al., 2006). Aside from this, the prevalence of IUD among adolescents is much higher in Asian countries (2% to 18%) than in European countries (1%–9%; Spada, 2014). See also a recent editorial with prevalences for China (Montag & Becker, 2020). These observations underline that both genetic and environmental factors are of relevance to be considered in the study of autistic traits and IUD tendencies.

**FIGURE 1:**
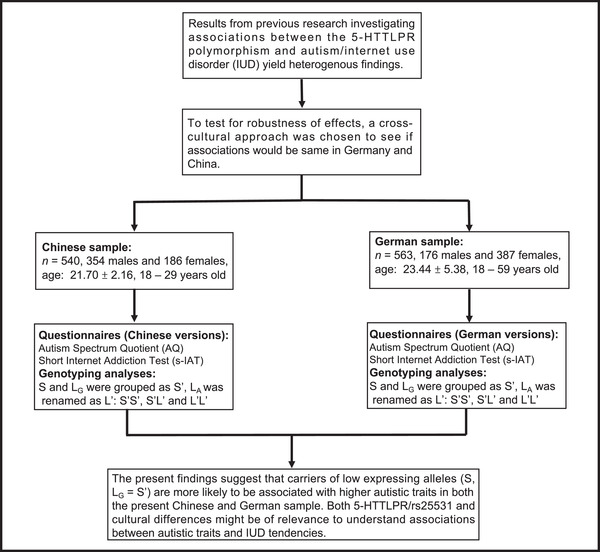
Flow chart of the research process of this study

In the current study, we investigated the potential associations between 5‐HTTLPR/rs25531 and autistic traits as well as IUD tendencies in subclinical populations from two countries with Asian and Caucasian populations (China and Germany; see also Figure 1 with the flowchart of the main steps in the present study). Given that in the literature a role of sex has been found to be of relevance in genetic association studies of 5‐HTTLPR (Laplante et al., 2019), we also took into account sex as a relevant variable in our statistical analyses. To sum up: Firstly, we revisited findings from our last work with a focus on the investigation of positive relations between autistic traits and IUD tendencies (Zhang et al., 2021). Secondly, we were interested to explore associations of 5‐HTTLPR/rs25531 with individual differences in autistic traits and IUD tendencies, and to assess whether the relationship between autistic traits and IUD tendencies varies by this genetic marker in participants from China and Germany. We expected that (1) higher autistic traits should be correlated with higher tendencies toward IUD in both samples; (2) certain constellations of 5‐HTTLPR/rs25531 (probably the S’ allele, although findings are heterogenous) might be a genetic risk factor for developing autistic traits and IUD tendencies and might affect the associations between autistic traits and IUD tendencies, potentially to a different extent in the Chinese and German samples. In particular, the latter part of the hypothesis section (2) is of an exploratory nature.

## 2 MATERIALS AND METHODS

### 2.1 Participants

A total of *N* = 1103 participants (540 Chinese, 563 German) were recruited in the present study. Participants gave written informed consent to participate in our study and received monetary compensation. The procedure was mostly identical in China and Germany, including protocols for questionnaires and 5‐HTTLPR/rs25531 genotyping (see for some differences in the s‐IAT measures section below). As the aim was to detect effects at a subclinical level, only participants who reported having no history of neurological or psychiatric disorders were included in the study. The present study focused on autistic traits; participants with diagnosed autism disorder were not considered in the present analyses.

In the Chinese sample, participants were included from two separate projects. Some participants (*n* = 304, age: 21.73 ± 2.03,18–27 years; sex: 230 males vs. 74 females) were recruited as part of the Chengdu Gene Brain Behavior Project (CGBBP) and the remaining (*n* = 236, age: 21.65 ± 2.33, 18–29 years; 124 males vs. 112 females) were recruited from an experiment measuring psychological traits outside the CGBBP at University of Electronic Science and Technology of China (UESTC). In the German sample, all participants (*n* = 563, age: 23.44 ± 5.38, 18–59 year; sex: 176 males vs. 387 females) were recruited via the Ulm Gene Brain Behavior Project (UGBBP) at Ulm University. A subgroup of the here‐analyzed participants from CGBBP in the Chinese sample (*n* = 166) has been included in our last work investigating the relationship between autistic traits, internet literacy, and IUD (Zhang et al., 2021). Of note, there is no overlap with the present German sample and the one reported in Zhang et al. (2021). As the main purpose of the current study was to assess the molecular genetic associations between 5‐HTTLPR/rs25531 and autistic traits/IUD tendencies, the overlapping subjects were kept in the Chinese sample to enhance the statistical power. Moreover, we also ran the same statistical analyses for assessing associations between autistic traits and IUD tendencies after excluding the overlapping participants, and these results are presented in the Supplementary Materials (from Supplementary Table 4 to Supplementary Table 7). The current study was approved by the local ethics committees at UESTC (number: neuscan‐298/302), Chengdu, China as well as at Ulm University, Ulm, Germany (number: 127/13).

### 2.2 Self‐report measures

To measure individual differences in the levels of autistic traits and the tendencies toward IUD, two validated self‐report questionnaires, that is, autism spectrum quotient (AQ) (Baron‐Cohen et al., 2001) and the short internet addiction test (s‐IAT) (Stodt et al., 2018) were assessed via filling in the survey on the online platform SurveyCoder programmed by Christopher Kannen (ckannen.com). Both questionnaires are available in Chinese and German.

The AQ was used to measure the level of autistic traits in populations with normal intelligence (Baron‐Cohen et al., 2001). It consists of 50 items and each item is a brief statement answered via a 4‐point Likert response (from “1 = definitely agree” to “4 = definitely disagree”). Response to each item is coded using a binary system (0/1), where an endorsement of the autistic trait (either “slightly agree”, or “definitely agree”) is scored as 1, while other responses are scored as 0. Total scores of the AQ range from 0 to 50 with higher scores reflecting the presence of more severe symptoms of autistic traits (Ruzich et al., 2015; Woodbury‐Smith et al., 2005). The original version of AQ was designed to yield a five factor structure (10 questions pertaining to each of the five subscales) including *social skills*, *attention switching*, *attention to details*, *communication*, and *imagination* (Baron‐Cohen et al., 2001). However, internal consistencies of some subscales showed low reliability coefficients in both samples (e.g., *attention switching* and *imagination* subscales, Cronbach's *α* < 0.500, see Table 1) and we therefore used another AQ subscale classification proposed by Austin (2005) constituting three subscales: *social skills*, *details/pattern*, and *communication/mindreading*. This version includes 26 items selected from the original 50 items and yielded better internal consistencies in both samples as presented in Table 1 (see reliabilities for both the five and three subscale structure versions in this table). In order to be more precise: we report data for the total AQ scores (stemming from the 50 item version: “AQ scores”) and the shortened version. This means “small AQ scores” in the present study.

**TABLE 1:** The reliabilities (Cronbach's *α*) of variables under investigation in the Chinese and German samples

	Chinese sample	German sample
Domain/variable	(*n* = 540)	(*n* = 563)
AQ scores	0.669	0.762
AQ SS	0.703	0.735
AQ AS	0.278	0.472
AQ AD	0.470	0.586
AQ CO	0.555	0.584
AQ IM	0.400	0.463
Small AQ scores	0.558	0.725
AQ SS	0.705	0.762
AQ DP	0.494	0.581
AQ CM	0.537	0.508
s‐IAT	0.889	0.832
s‐IAT LoC/TM	0.828	0.781
s‐IAT C/SP	0.800	0.771

Abbreviations: AQ, the Adult Autism Spectrum Quotient; SS, social skills; AS, attention switching; AD, attention to details; CO, communication; IM, imagination; DP, details/pattern; CM, communication/mindreading; s‐IAT, the short Internet Addiction Test; LoC/TM, loss of control/time management; C/SP, craving/social problems.

The s‐IAT consists of 12 items to assess the level of problematic internet use based on a 5‐point Likert scale (from “1 = rarely” to “5 = very often”) and has two subscales *loss of control/time management* and *craving/social problems* representing key elements of IUD. Total scores of the s‐IAT can range from 12 to 60 and higher scores indicate more severe problems due to one's own internet use. The s‐IAT shows good psychometric properties (see Table 1). The s‐IAT used in Germany was the version from the internet addiction test with 20 items (Young, 1998) and was cut to 12 items. This is not a problem though, because the content of the items is comparable with the s‐IAT (Stodt et al., 2018), but they differ slightly in each item response (from “1 = rarely” to “5 = always”).

### 2.3 DNA extraction and genotyping analyses

DNA was automatically extracted from buccal cells by means of a MagNA Pure 96 robot system with a commercial extraction kit from Roche (Mannheim, Germany). Genotyping of 5‐HTTLPR/rs25531 was conducted via polymerase chain reaction (PCR) with subsequent digestion and gel electrophoresis. The following primers (TIB MOLBOL, Berlin, Germany) for PCR were used (Wendland et al., 2006): the forward primer (5‐HTT‐MSP_F): 5′‐TCCTCCGCTTTGGCGCCTCTTCC−3′ and the reverse primer (5‐HTT‐MSP_R): 5′‐TGGGGGTTGCAGGGGAGATCCTG−3′. The following PCR program was used: 30s at 95°C, 30s at 64,9°C, 60s at 72°C for 35 cycles with an initial denaturation step of 5 min at 95°C and a final elongation step of 5min at 72°C. To identify the A/G SNP rs25531 regarding the L allele, the PCR‐product was digested with the restriction endonuclease MspI (restriction enzyme, New England Biolabs) and incubated at 37°C for 1.5 h. Thereafter, samples were loaded onto a 1.8% agarose gel in a TBE solution, running for 2 h at 175 V. After completion of the gel electrophoresis, results were documented by the means of a gel documentation system with UV light. At the end of the process, samples were visualized and classified by two independent raters. Due to the low frequency of the 5‐HTTLPR/rs25531 extra‐long (XL) allele (18 repeats) as well as extra‐extra‐long (XXL) allele (XXL, 20 repeats) (Lesch et al., 1997) in both samples (XL: China *n* = 19; XXL: China *n* = 4 vs. Germany *n* = 1), these were treated as L_A_ alleles in the subsequent analyses.

### 2.4 Statistical analyses

Statistical analyses were performed with the SPSS 26.0 software (IBM Corp, Armonk, NY, USA). Before carrying out the main statistical analyses, the distributions of all variables under investigation were tested for normality separately in the Chinese and German samples (see Supplementary Table 1). As AQ scores were normally distributed in both samples, whereas s‐IAT scores were not in the German sample, parametric tests were used for AQ scores and nonparametric tests were used for s‐IAT scores in both samples to make the analyses comparable between the Chinese and German samples (but findings from both parametric and non‐parametric tests for s‐IAT scores are presented).

Allele and genotype frequencies of 5‐HTTLPR/rs25531 were calculated, and genotypic distributions were tested for Hardy–Weinberg equilibrium separately in the Chinese and German samples. In order to check if in the main analyses age was needed to be controlled for, the following analyses were performed: Pearson correlations between age and AQ scores as well as its subscales were calculated. Additionally, Spearman correlations were used to examine age effects on s‐IAT scores as well as its subscales, respectively in the Chinese and German samples.

With respect to the genetic data analyses, subjects were classified regarding the 5‐HTTLPR/rs25531 genetic information depending on the transcriptional activities of 5‐HTTLPR/rs25531 as described in Zalsman et al. (2006): both the S and L_G_ alleles were grouped/named as the S’ allele. Therefore, groups of S'S’ (low expressing allele homozygosity), S'L’ (intermediate expressing allele heterozygosity), and L'L’ (high expressing allele homozygosity) were formed. Despite a small number of L'L’ carriers in the Chinese sample, we implemented the same grouping (S'S’ vs. S'L’ vs. L'L’) in the two samples in order to facilitate direct comparisons between the Chinese and German samples.

To revisit the findings from Zhang et al. (2021) in our present dataset, relationships between AQ scores and s‐IAT scores were calculated using partial correlations, controlling for age and sex separately in the Chinese and German samples. Fisher's Z tests were used to assess correlation differences between the Chinese and German samples. Furthermore, correlations between AQ scores and s‐IAT scores were also investigated in different 5‐HTTLPR/rs25531 genotype groups, again separately in the Chinese and German samples, which then could be contrasted.

To test whether potential associations between autistic traits/IUD tendencies might be modulated by 5‐HTTLPR/rs25531 and sex, ANCOVAs with genotypes [S'S’ vs. S'L’ vs. L'L’] and sex (male vs. female) as fixed factors (independent variables) and age as covariate were conducted with mean‐scores of AQ and s‐IAT (after the square root‐transformation, we performed this step for the s‐IAT to improve the normality of skewed distributions (Tabachnick & Fidell, 1996)) being the dependent variables in both Chinese and German samples. For follow‐up tests, we used Bonferroni corrected post‐hoc tests in SPSS when relevant (these are presented as ps). With this option, SPSS returns *p*–values that are Bonferroni corrected (see IBM support, the calculation of Bonferroni‐adjusted *p*‐values)^1^. Results were considered significant at the p/ps < 0.05 level.

## 3 RESULTS

### 3.1 Demographics of 5‐HTTLPR/rs25531 and questionnaires

As indicated in the previous section, 5‐HTTLPR/rs25531 data were analyzed following the tri‐allelic classification (see the work by Zalsman et al. (2006)). Frequencies of 5‐HTTLPR/rs25531 alleles and genotypes for both Chinese and German samples are presented in Table 2. In both samples, distributions of 5‐HTTLPR/rs25531 genotypes were in Hardy–Weinberg equilibrium (China: *χ* ^2^ = 0.19, *df* = 1, *p* = 0.665; Germany: *χ* ^2^ = 0.01, *df* = 1, *p =* 0.907). The demographic statistics of questionnaires under investigation according to sex and 5‐HTTLPR/rs25531 genotypes are presented in Table 3 for the Chinese and German cohorts separately.

**TABLE 2:** Frequencies of 5‐HTTLPR/rs25531 alleles and genotypes in the Chinese and German samples

	Chinese sample		German sample	
	(*n* = 540)		(*n* = 563)	
Genotypes	Frequency	Percentage (%)	Frequency	Percentage (%)
S'S’	357	66.11	118	20.96
S'L’	166	30.74	281	49.91
L'L’	17	3.15	164	29.13
Alleles				
S	777	71.94	438	38.90
L_A_	176	16.30	608	54.00
L_G_	103	9.54	79	7.01
XL	20	1.85	0	0.00
XXL	4	0.37	1	0.09

S'S’ (low expressing allele homozygosity): S/S, S/L_G_ L_G_/L_G_; S'L’ (intermediate expressing allele heterozygosity): L_A_/S, L_A_/L_G_ XL/S, XL/L_G_, XXL/S, XXL/L_G_; L'L’ (high expressing allele homozygosity): L_A_/L_A,_ XL/L_A,_ XL/XL.

**TABLE 3:** Demographic and questionnaire scores in the Chinese and German samples according to sex and 5‐HTTLPR/rs25531 genotypes

		**Sex**			**5‐HTTLPR/rs25531 genotype**			
**Variables**	**Total sample**	**Males**	**Females**	***p1***	**S'S’**	**S'L’**	**L'L’**	***p2***
**Chinese sample**	(*n* = 540)	(*n* = 354)	(*n* = 186)		(*n* = 357)	(*n* = 166)	(*n* = 17)	
Age	21.70 (2.16)	21.75 (2.22)	21.60 (2.05)	*0.455*	21.68 (2.13)	21.72 (2.23)	21.88 (2.37)	*0.916*
AQ total	21.97 (5.75)	22.32 (5.71)	21.30 (5.77)	*0.050*	22.30 (5.79)	21.36 (5.63)	21.06 (5.79)	*0.174*
small AQ	11.60 (3.60)	11.87 (3.56)	11.09 (3.62)	*0.016*	11.95 (3.55)	11.00 (3.66)	10.24 (3.09)	*0.005*
AQ SS	4.96 (2.83)	5.04 (2.85)	4.83 (2.79)	*0.415*	5.15 (2.77)	4.62 (2.90)	4.35 (3.06)	*0.088*
AQ DP	4.49 (1.80)	4.67 (1.83)	4.16 (1.70)	*0.002*	4.58 (1.82)	4.34 (1.74)	4.12 (1.96)	*0.257*
AQ CM	2.14 (1.56)	2.17 (1.60)	2.10 (1.48)	*0.648*	2.21 (1.54)	2.04 (1.63)	1.76 (1.30)	*0.288*
s‐IAT total	33.03 (8.64)	33.24 (8.95)	32.62 (8.02)	*0.453*	32.76 (8.51)	33.52 (8.85)	33.71 (9.43)	*0.945*
s‐IAT LoC/TM	18.14 (4.65)	17.95 (4.72)	18.52 (4.52)	*0.195*	18.02 (4.66)	18.41 (4.68)	18.12 (4.40)	*0.847*
s‐IAT C/SP	14.88 (4.62)	15.29 (4.83)	14.10 (4.07)	*0.004*	14.74 (4.49)	15.11 (4.82)	15.59 (5.29)	*0.873*
**German sample**	(n = 563)	(n = 176)	(n = 387)		(n = 118)	(n = 281)	(n = 164)	
Age	23.44 (5.38)	24.83 (6.58)	22.80 (4.61)	*< 0.001*	23.50 (6.27)	23.24 (4.77)	23.73 (5.69)	*0.648*
AQ total	16.30 (6.02)	18.27 (6.13)	15.41 (5.76)	*< 0.001*	17.17 (6.84)	16.49 (6.02)	15.35 (5.26)	*0.033*
small AQ	8.29 (3.92)	9.59 (4.08)	7.70 (3.71)	*< 0.001*	8.82 (4.28)	8.41 (4.03)	7.70 (3.38)	*0.045*
AQ SS	2.75 (2.59)	3.39 (2.92)	2.47 (2.37)	*< 0.001*	3.07 (2.76)	2.78 (2.60)	2.48 (2.43)	*0.168*
AQ DP	4.52 (1.93)	5.08 (1.90)	4.26 (1.89)	*< 0.001*	4.53 (1.87)	4.63 (1.93)	4.31 (1.96)	*0.248*
AQ CM	1.02 (1.19)	1.12 (1.27)	0.97 (1.15)	*0.189*	1.22 (1.23)	1.00 (1.20)	0.90 (1.14)	*0.081*
s‐IAT total	17.57 (5.21)	18.32 (5.72)	17.23 (4.93)	*0.023*	17.76 (4.86)	17.47 (5.53)	17.59 (4.92)	*0.379*
s‐IAT LoC/TM	10.58 (3.75)	10.86 (3.92)	10.46 (3.66)	*0.241*	10.75 (3.68)	10.45 (3.87)	10.70 (3.59)	*0.352*
s‐IATC/SP	6.98 (2.08)	7.46 (2.50)	6.77 (1.82)	*< 0.001*	7.01 (1.67)	7.02 (2.27)	6.90 (2.00)	*0.300*

Abbreviations: AQ, the Adult Autism Spectrum Quotient; SS, social skills; DP, details/pattern; CM, communication/mindreading; s‐IAT, the short Internet Addiction Test; LoC/TM, loss of control/time management; C/SP, craving/social problems. S’S’ (low expressing allele homozygosity): S/S, S/L_G_ L_G_/L_G_; S’L’ (intermediate expressing allele heterozygosity): L_A_/S, L_A_/L_G_ XL/S, XL/L_G_, XXL/S, XXL/L_G_; L’L’ (high expressing allele homozygosity): L_A_/L_A_, XL/L_A_, XL/XL; p1 = refers to the comparison of males and females, p2 = refers to the comparison of different genotypes.

Age was found to significantly correlate with scores of AQ total and AQ subscales in the German sample, as well as s‐IAT total and s‐IAT subscales in both samples (details are presented in the Supplementary Material). Hence, age was included as a control variable in subsequent analyses.

### 3.2 Associations between autistic traits and IUD tendencies in the Chinese and German samples

Firstly, in order to revisit earlier findings (Zhang et al., 2021) in our present dataset, we used parametric partial correlations, controlling for sex and age (as the s‐IAT was not normally distributed, results of non‐parametric partial correlations are presented in Supplementary Table 2 and were comparable to the results from parametric tests). Results are presented in Table 4. The correlation patterns were rather similar in both samples. More specifically, AQ total scores, small AQ scores, AQ *social skill* scores, and AQ *communication/mindreading* scores were positively correlated with s‐IAT total and subscale scores in both samples, whereas scores of the AQ *details/pattern* subscale were negatively correlated with s‐IAT total and subscale scores, for statistical significance see Table 4. Fisher’ *Z*‐tests indicated that correlation strengths differed significantly between the German and the Chinese sample for correlations of AQ *social skills*/s‐IAT total (*p* = 0.031), small AQ/s‐IAT *craving/social problems* (*p* = 0.030), AQ *social skills*/s‐IAT *craving/social problems* (*p* = 0.006), and AQ *communication/mindreading*/s‐IAT *craving/social problems* (*p* = 0.049) (lower in the Chinese sample compared to German sample).

**TABLE 4:** Partial correlations between AQ and s‐IAT scores in the Chinese and German samples, controlled for sex and age

	Chinese sample			German sample			Fisher's *Z*		
	(*n* = 540)			(*n* = 563)					
Variables	s‐IAT total	s‐IAT LoC/TM	s‐IAT C/SP	s‐IAT total	s‐IAT LoC/TM	s‐IAT C/SP	s‐IAT total	s‐IAT LoC/TM	s‐IAT C/SP
AQ total	0.17***	0.12**	0.19***	0.20***	0.13**	0.27***	−0.65	−0.17	−1.49
small AQ	0.11*	0.06	0.14***	0.22***	0.16***	0.27***	−1.93	−1.64	−2.17*
AQ SS	0.08	0.05	0.10*	0.20***	0.14***	0.26***	−2.15*	−1.57	−2.73**
AQ DP	−0.09*	−0.09*	−0.08	−0.02	−0.02	−0.03	−1.14	−1.24	−0.89
AQ CM	0.22***	0.17***	0.24***	0.32***	0.25***	0.35***	−1.77	−1.39	−1.97*

Abbreviations: AQ, the Adult Autism Spectrum Quotient; SS, social skills; DP, details/pattern; CM, communication/mindreading; s‐IAT, the short Internet Addiction Test; LoC/TM, loss of control/time management; C/SP, craving/social problems.

* *p* < 0.05.

** *p* < 0.01.

*** *p* < 0.001.

### 3.3 Associations between autistic traits and IUD tendencies according to 5‐HTTLPR/rs25531 genotypes in the Chinese and German samples

Further analyses of the (positive) AQ/s‐IAT correlations for each genotype group showed that in the whole Chinese sample, they were mainly driven by S'S’ and S'L’ carriers, while in the whole German sample by S'L’ carriers, followed by L'L’ carriers (see Table 5). Moreover, Fisher’ *Z* tests revealed significant differences between S'S’ carriers of the two samples regarding correlation strengths of AQ total/s‐IAT total (*p* = 0.041) and AQ total/s‐IAT *loss of control/time management* (*p* = 0.021). Significantly different correlation strengths between small AQ and s‐IAT total/*loss of control/time management* scores, between AQ social skills and s‐IAT total/*loss of control/time management* scores, and between AQ *communication/mindreading* and all s‐IAT total and subscale scores were found in S'L’ carriers of the German compared to those of the Chinese sample (all *p* ≤ 0.043). For L'L’ carriers, correlation strengths between scores of small AQ and s‐IAT *craving/social problems* and between AQ *social skills* and all s‐IAT total scores were also significantly different in the German compared to the Chinese sample (all *p* ≤ 0.042).

**TABLE 5:** Partial correlations between AQ and s‐IAT scores in the Chinese and German samples by 5‐HTTLPR/rs25531 genotypes, controlled for sex and age

	Chinese sample			German sample			Fisher's *Z*		
Variables	s‐IAT total	s‐IAT LoC/TM	s‐IAT C/SP	s‐IAT total	s‐IAT LoC/TM	s‐IAT C/SP	s‐IAT total	s‐IAT LoC/TM	s‐IAT C/SP
S'S’	(*n* = 357)			(*n* = 118)					
AQ total	0.18***	0.15**	0.19***	−0.04	−0.10	0.11	2.05*	2.32*	0.71
small AQ	0.14**	0.09	0.17**	0.01	−0.04	0.11	1.23	1.19	0.58
AQ SS	0.10	0.08	0.11*	0.02	−0.01	0.09	0.78	0.90	0.19
AQ DP	−0.11*	−0.13*	−0.08	−0.16	−0.19*	−0.07	0.46	0.51	−0.13
AQ CM	0.27***	0.21***	0.29***	0.23*	0.18*	0.27**	0.37	0.30	0.14
**S'L’**	(n = 116)		(n = 281)						
AQ total	0.20*	0.13	0.24**	0.30***	0.25***	0.31***	−1.12	−1.24	−0.79
small AQ	0.12	0.07	0.15	0.32***	0.28***	0.31***	−2.17*	2.19*	−1.69
AQ SS	0.11	0.06	0.14	0.31***	0.27***	0.31***	−2.18*	−2.14*	−1.80
AQ DP	−0.07	−0.04	−0.09	0.02	0.04	−0.02	−0.97	−0.87	−0.76
AQ CM	0.15	0.09	0.18*	0.36***	0.28***	0.39***	−2.29*	−2.02*	−2.29*
**L'L’**	(*n* = 17)		(*n* = 164)						
AQ total	−0.16	−0.19	−0.13	0.19*	0.09	0.30***	−1.29	−1.00	−1.58
small AQ	−0.22	−0.16	−0.26	0.19*	0.09	0.29***	−1.48	−0.91	−2.03*
AQ SS	−0.46	−0.52*	−0.36	0.13	0.02	0.27***	−2.24*	−2.16*	−2.33*
AQ DP	0.23	0.39	0.06	−0.02	−0.01	−0.03	0.90	1.49	0.30
AQ CM	0.20	0.25	0.13	0.30***	0.22**	0.33***	−0.38	0.10	−0.74

Abbreviations: AQ, the Adult Autism Spectrum Quotient; SS, social skills; DP, details/pattern; CM, communication/mindreading; s‐IAT, the short Internet Addiction Test; LoC/TM, loss of control/time management; C/SP, craving/social problems. S’S’ (low expressing allele homozygosity): S/S, S/L_G_ L_G_/L_G_; S’L’ (intermediate expressing allele heterozygosity): L_A_/S, L_A_/L_G_ XL/S, XL/L_G_, XXL/S, XXL/L_G_; L’L’ (high expressing allele homozygosity): L_A_/L_A_, XL/L_A_, XL/XL.

* *p* < 0.05.

** *p* < 0.01.

*** *p* < 0.001.

### 3.4 Effects of 5‐HTTLPR/rs25531 and sex on autistic traits

**FIGURE 2:**
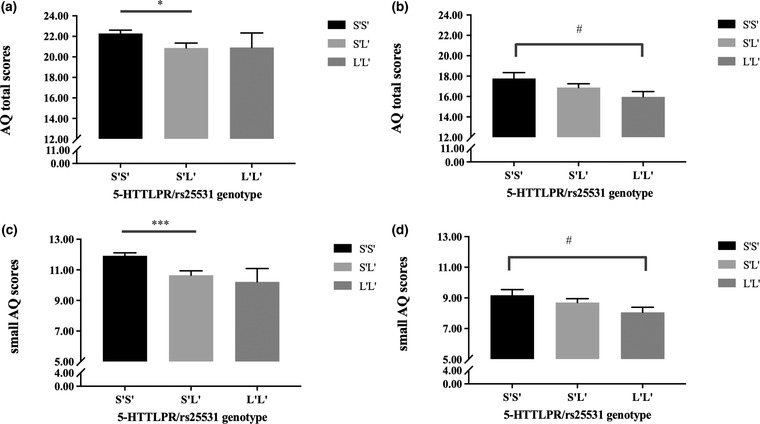
Mean scores of AQ total (top) and small AQ (down) in 5‐HTTLPR/rs25531 genotype groups presented separately in the Chinese and German samples (as derived by the ANCOVA, therefore the depicted results slightly differ from the information in Table 3). (a) and (c) for the Chinese sample, (b) and (d) for the German sample. Error bars show standard errors. ^*^ *p* < 0.05, ^***^ *p* < 0.001, ^#^ *p* < 0.1

**FIGURE 3:**
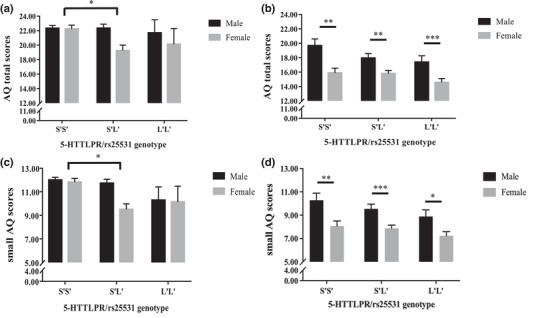
Mean scores of AQ total (top) and small AQ (down) for males and females in 5‐HTTLPR/rs25531 genotype groups presented separately in the Chinese and German samples (as derived by the ANCOVA (therefore the depicted results slightly differ from the information in Table 3). (a) and (c) for the Chinese sample, (b) and (d) for the German sample. Error bars show standard errors. ^*^ *p* < 0.05, ^**^ *p* < 0.01, ^***^ *p* < 0.001

A summary of the results is presented in Table 6. In the Chinese sample, ANCOVAs revealed a significant main effect of 5‐HTTLPR/rs25531 on the AQ total scores (*p* = 0.037), small AQ scores (*p* < 0.001), and AQ social skills (*p* = 0.025). Post‐hoc analyses revealed significantly higher AQ total scores in S'S’ homozygotes compared to S'L’ carriers (AQ total, S'S’: 22.30 ± 5.79 vs. S'L’: 21.36 ± 5.63, corrected *ps* = 0.039, see Figure 2a), also higher small AQ scores (S'S’: 11.95 ± 3.55 vs. S'L’: 11.00 ± 3.66, corrected *ps* < 0.001, see Figure 2c) and AQ *social skills* scores (S'S’: 5.15 ± 2.77 vs. S'L’: 4.62 ± 2.90, corrected *ps* = 0.027). Please note that the L'L’ group is very small (*n* = 17) and therefore it is hard to investigate this group in the Chinese sample (please note that descriptively, the L'L’ group in the Chinese sample showed the lowest AQ scores). The main effect of sex was significant on the AQ *details/pattern* subscale (*p* = 0.044), with males having significantly higher scores relative to females (male: 4.67 ± 1.83 vs. female: 4.16 ± 1.70, corrected *ps* = 0.044). Importantly, a significant interaction effect between 5‐HTTLPR/rs25531 and sex was found on AQ total scores (*p* = 0.041) and small AQ scores (*p* = 0.018). Post‐hoc analyses showed significantly higher AQ total scores (S'S’: 22.26 ± 5.76 vs. S'L’: 19.39 ± 5.33, corrected *ps* = 0.006, see Figure 3a) and small AQ scores (S'S’: 11.83 ± 3.43 vs. S'L’: 9.57 ± 3.56, corrected *ps* < 0.001, see Figure 3c) in females with S'S’ homozygote compared to S'L’ carriers, but no significant difference between males with S'S’ homozygote and S'L’ carriers (corrected *ps* > 0.406). In sum, the genetic effect of 5‐HTTLPR/rs25531 on the AQ variables was driven by females in the Chinese sample.

**TABLE 6:** Genotypes and sex: Main effects and interactions on AQ scores and its subscale scores in the Chinese and German samples

	Chinese sample			German sample		
	(*n* = 540)			(*n* = 563)		
AQ Scores	*F*	*p*	*η^2^*	*F*	*p*	*η^2^*
**AQ total**						
5‐HTTLPR (S'S’ vs. S'L’ vs. L'L’)	3.31	*0.037*	0.012	2.78	*0.063*	0.010
Sex (M vs. F)	2.32	*0.129*	0.004	25.46	*< 0.001*	0.044
5‐HTTLPR × Sex	3.22	*0.041*	0.012	0.74	*0.478*	0.003
**small AQ**						
5‐HTTLPR (S'S’ vs. S'L’ vs. L'L’)	7.61	*< 0.001*	0.028	2.58	*0.077*	0.009
Sex (M vs. F)	1.76	*0.186*	0.003	23.99	*< 0.001*	0.041
5‐HTTLPR × Sex	4.03	*0.018*	0.015	0.21	*0.814*	< 0.001
**AQ SS**						
5‐HTTLPR (S'S’ vs. S'L’ vs. L'L’)	3.71	*0.025*	0.014	1.62	*0.198*	0.006
Sex (M vs. F)	0.05	*0.826*	< 0.001	12.24	*< 0.001*	0.022
5‐HTTLPR × Sex	2.61	*0.074*	0.010	0.25	*0.777*	< 0.001
**AQ DP**						
5‐HTTLPR (S'S’ vs. S'L’ vs. L'L’)	2.18	*0.114*	0.008	1.57	*0.209*	0.006
Sex (M vs. F)	4.07	*0.044*	0.008	16.67	*< 0.001*	0.029
5‐HTTLPR × Sex	1.47	*0.230*	0.006	0.62	*0.539*	0.002
**AQ CM**						
5‐HTTLPR (S'S’ vs. S'L’ vs. L'L’)	1.35	*0.260*	0.005	2.49	*0.084*	0.009
Sex (M vs. F)	0.09	*0.760*	< 0.001	3.01	*0.083*	0.005
5‐HTTLPR × Sex	0.11	*0.898*	< 0.001	1.54	*0.215*	0.006

Abbreviations: AQ, the Adult Autism Spectrum Quotient; SS, social skills; DP, details/pattern; CM, communication/mindreading. M, male, F, female. S’S’ (low expressing allele homozygosity): S/S, S/L_G_ L_G_/L_G_; S’L’ (intermediate expressing allele heterozygosity): L_A_/S, L_A_/L_G_ XL/S, XL/L_G_, XXL/S, XXL/L_G_; L’L’ (high expressing allele homozygosity): L_A_/L_A_, XL/L_A_, XL/XL.

In the German sample, there was a trend toward a significant main effect of 5‐HTTLPR/rs25531 on AQ total scores (*p* = 0.063) and on small AQ scores (*p* = 0.077). Exploratory post‐hoc analyses revealed marginally significantly higher AQ total scores in S'S’ homozygote compared to L'L’ carriers (AQ total, S'S’: 17.17 ± 6.84 vs. L'L’: 15.35 ± 5.26, corrected *ps* = 0.057, see Figure 2b), and small AQ scores (S'S’: 8.82 ± 4.28 vs. L'L’: 7.70 ± 3.38, corrected *ps* = 0.078, see Figure 2d). Significant main effects of sex were found on AQ scores as well as on all the subscales (*p* < 0.001) except the *communication/mindreading* subscale (*p* = 0.083), with significantly higher AQ or AQ subscale scores in males than females (see Figure 3b,d). However, there was no significant interaction between 5‐HTTLPR/rs25531 and sex (see Table 6).

### 3.5 Effects of 5‐HTTLPR/rs25531 and sex on IUD tendencies

ANCOVAs showed no significant main effect of 5‐HTTLPR/rs25531 and no significant interaction between 5‐HTTLPR/rs25531 and sex on s‐IAT scores and on s‐IAT subscale scores either in the Chinese or German sample. Several main effects of sex were found in both samples: In the Chinese sample, the main effect of sex on s‐IAT *craving/social problems* (*p* = 0.014) was significant. In the German sample, significant main effects of sex on s‐IAT total scores as well as both subscales were found (*p* ≤ 0.039) (see Table 7), with significantly higher s‐IAT scores in males than females.

**TABLE 7:** Genotypes and sex: Main effects and interactions on s‐IAT scores and its subscale scores the Chinese and German samples

	**Chinese sample**			**German sample**		
	(n = 540)			(n = 563)		
**s‐IAT Scores**	F	*p*	η^2^	F	*p*	η^2^
**s‐IAT total**						
5‐HTTLPR (S'S’ vs. S'L’ vs. L'L’)	0.61	*0.541*	0.002	0.82	*0.443*	0.003
Sex (M vs. F)	1.60	*0.206*	0.003	9.38	***0.002***	0.017
5‐HTTLPR × Sex	0.79	*0.454*	0.003	0.96	*0.382*	0.003
**s‐IAT LoC/TM**						
5‐HTTLPR (S'S’ vs. S'L’ vs. L'L’)	0.71	*0.493*	0.003	1.36	*0.258*	0.005
Sex (M vs. F)	0.00	*0.984*	< 0.001	4.30	***0.039***	0.008
5‐HTTLPR × Sex	0.80	*0.450*	0.003	0.95	*0.387*	0.003
**s‐IAT C/SP**						
5‐HTTLPR (S'S’ vs. S'L’ vs. L'L’)	0.46	*0.630*	0.002	0.09	*0.914*	< 0.001
Sex (M vs. F)	6.03	***0.014***	0.011	16.53	***< 0.001***	0.029
5‐HTTLPR × Sex	0.77	*0.463*	0.003	0.53	*0.589*	0.002

Abbreviations: s‐IAT, the short Internet Addiction Test; LoC/TM, loss of control/time management; C/SP, craving/social problems. M, male, F, female. S’S’ (low expressing allele homozygosity): S/S, S/L_G_ L_G_/L_G_; S’L’ (intermediate expressing allele heterozygosity): L_A_/S, L_A_/L_G_ XL/S, XL/L_G_, XXL/S, XXL/L_G_; L’L’ (high expressing allele homozygosity): L_A_/L_A_, XL/L_A_, XL/XL.

## 4 DISCUSSION

Using the tri‐allelic classification when investigating the 5‐HTTLPR/rs25331 complex, the present study investigated whether autistic traits, IUD tendencies, and their associations with each other varied as a function of 5‐HTTLPR/rs25531 in two independent samples with a different cultural background (China and Germany). Results showed that association patterns between autistic traits and IUD tendencies in the three 5‐HTTLPR/rs25531 genotype groups differed between the Chinese and German samples. Dissociative effects of 5‐HTTLPR/rs25531 on autistic traits between males and females were observed but no significant effects on IUD tendencies either in the Chinese or the German samples appeared. But first, we would like to discuss the most consistent findings, namely of 5‐HTTLPR/rs25531 on autistic traits in both German and Chinese investigated samples.

A different distribution of 5‐HTTLPR/rs25531 between the Chinese sample (S'S’: 66.1%; S'L’: 30.7%; L'L’: 3.1%) and the German sample (S'S’: 21.0 %; S'L’: 49.9 %; L'L’: 29.1%) was found in the present study, and the genotype distribution for each nation was consistent with the results reported in previous studies (Ho et al., 2016; Zalsman et al., 2006). Furthermore, in accordance with our hypothesis in the Chinese sample, the present study found that carriers of the low expressing S'S’ genotype had highest levels of autistic traits depending on the scores of small AQ and AQ *social skills* subscale, followed by carriers of intermediate expressing S'L’, and then carriers of high expressing L'L’ genotype. The German sample showed a similar pattern, although the main effect of 5‐HTTLPR/rs25531 was only marginally significant (we refrained from heightening statistical power by testing directed hypotheses, which could have been done in line with our a priori hypothesis). The 5‐HTTLPR‐findings are consistent with a previous study with ASD patients demonstrating that the S allele (but not including the SNP rs25531 in the analysis) was associated with more severe impairments in the communication and social interaction domains of the AQ and also being a genetic risk factor for developing ASD (Tordjman et al., 2001). Nevertheless, a second look at the data shows that some of our findings are more complex than so far described. Mounting evidence suggests that the serotonergic system is differentially regulated in male and female individuals, such as different levels of serotonin metabolism (females > males) (Lu et al., 2016), and rates of serotonin synthesis (males > females) (Nishizawa et al., 1997; Sakai et al., 2006) that are largely determined by the function of 5‐HTT. A previous review also suggested that on molecular genetic level, 5‐HTTLPR variants may exert a differential modulation on affective disorders in the sexes (Gressier et al., 2016). They found the S allele to be associated with an increased risk of depression and anxiety among females and with an increased risk of aggressive, behavior conduct disorder, and higher externalizing behaviors among males. The current study also revealed a sex‐specific effect of 5‐HTTLPR/rs25531 on autistic traits in the Chinese sample, indicating that individuals with the low expressing alleles (S, L_G_) showed higher levels of autistic traits, but this effect varied by sex. More specifically, significantly higher scores of AQ total, small AQ, and the AQ *social skills* subscale were found in S'S’ than S'L’ carriers only in females but not in males. As mentioned in the introduction, the SS genotype and S allele of the bi‐allelic 5‐HTTLPR polymorphism have been put forward as a putative risk factor for autism in some works (Arieff et al., 2010b; Meguid et al., 2015). However, overall inconsistent results have been reported (Nonweiler et al., 2020; Wang et al., 2019; Wei et al., 2021). In the present study, using the tri‐allelic classification of 5‐HTTLPR/rs25531, we expected that not only the S allele was more closely linked to negative outcomes/negative emotionality as reported in some papers (Caspi et al., 2003; Juhasz et al., 2015; Lesch et al., 1996), but in the present work also that the investigated L_G_ allele as part of the S’ group (resembling the serotonin transporter activity of the S allele) from the tri‐allelic analysis would go along with higher autistic traits (please note that it was not possible to test the L_G_ allele effect alone). Taken together, investigating the effect of 5‐HTTLPR considering both the tri‐allelic classification and sex differences could enhance our understanding of the role of 5‐HTTLPR in ASD.

At this point, we also want to mention that in the German sample a clearer sex‐effect on autistic traits could be observed than in the Chinese sample; in particular, in the German sample, males showed on average significantly higher autistic traits than females. Previous research indicated that the expression of autistic traits could be influenced by ethnicity and cultural context of investigated individuals (Elhami Athar et al., 2021; Wakabayashi et al., 2006). To illustrate this: Freeth, Sheppard et al. (2013) observed that behaviors associated with autistic traits measured in neurotypical individuals were significantly higher in Eastern cultures (India and Malaysia) than in a Western culture (United Kingdom). In this paper, the authors speculate that Western societies with more person‐centered attributions for behavior compared to Eastern societies with more situation‐centered attributions probably result in different training of social skills in everyday life situations (Freeth, Sheppard et al., 2013). But much more research in this field is needed to find empirical evidence for sound explanations of this phenomenon. Beyond this, also sex differences in autistic traits measured by AQ are well described in the literature (Baron‐Cohen et al., 2001; Freeth, Bullock, et al., 2013; Ruzich et al., 2015; Zhao et al., 2019), indicating that males have higher autistic trait levels than females in the general population. A systematic review by Ruzich et al. (2015) came also to the conclusion that males on average have higher autistic trait scores than females in nonclinical populations, but such a difference could not be observed in the diagnosed autistic population (Ruzich et al., 2015). Taken together, both culture and sex are relevant variables to better understand autistic traits. And again, the cultural effects need to be more studied to come up with satisfying explanations.

Coming back to the molecular genetic findings, beyond the genetic associations with AQ scores, we did not find any significant modulatory effect of 5‐HTTLPR/rs25531 on IUD tendencies either in the Chinese or German samples, which was consistent with a recent study (Cerniglia et al., 2020). However, previous studies have reported that (male) individuals with the SS genotype showed increased risk to “Internet addiction” (Lee et al., 2008; Sun et al., 2016). Please note that we prefer the term IUD in line with recent developments in ICD‐11 (see Montag et al., 2021). Inconsistencies in the observed findings might be due to different methods of genetic data analyses (not considering the tri‐allelic classification), different questionnaires used, and different study populations.

For associations between autistic traits and IUD tendencies, the present study found similar patterns between the two samples that AQ total scores/small AQ and AQ subscales were positively correlated with s‐IAT total and subscale scores with the exception of the AQ *details/pattern* subscale, suggesting that individuals with higher autistic traits show greater tendencies toward developing IUD. This is basically a replication of what Zhang et al. (2021) observed in their recent work. Please note that results observed in Zhang et al. (2021) could also be replicated, when the overlapping participants between this older and the present study were excluded (see Supplementary Material). We also mention that statistical analysis taking into account the AQ facets according to the three subscale structure (Austin, 2005) showed a similar correlation pattern with the s‐IAT as in the original five facet structure (see Supplementary Table 3).

Summing up, we observed also in the present study that higher autistic traits go along with higher IUD tendencies, but effect sizes of the correlations are small (Ellis, 2010). This is in line with observations from previous studies (Finkenauer et al., 2012; Romano et al., 2014; Zhang et al., 2021). Importantly, although similar patterns were found between the complete Chinese and German samples, further analyses of putative correlation strength differences between the two samples revealed significantly stronger positive correlations between small AQ, AQ *social skills*, AQ *communication/mindreading* and s‐IAT *craving/social problems* in the German compared to the Chinese samples.

Besides, a further analysis focusing on the genetic variables (hence further subgrouping according to 5‐HTTLPR/rs25531 genotypes) revealed for the Chinese sample that the positive association between s‐IAT and AQ was driven by S'S’ and S'L' carriers and for the German sample, mainly driven by S'L’ and L'L' carriers. In line with patterns of correlation strength comparisons in the whole samples, correlations among S'L’ and L'L’ carriers between AQ scores and s‐IAT scores tended to be more positive in the German compared to the Chinese samples, although the specific significant correlation pairs were different. For S'S’ carriers, correlations were in an opposite way (more positive in the Chinese compared to the German samples). These findings provide further support for potential cultural differences between China and Germany regarding the relationship between autistic traits and IUD tendencies, and extend our previous study (Zhang et al., 2021). Gene‐culture coevolution theory has been proposed as an influential theory for explaining how behavior is a product of two‐complementary and interacting evolutionary processes by genetic and cultural evolution (Cavalli‐Sforza & Feldman, 1981; Chiao & Blizinsky, 2010; Richerson et al., 2010). For example, Ishii et al. (2014) observed that Japanese individuals with SS genotype detected the disappearance of happy facial expressions with greater perceptual efficiency than those with SL and LL genotypes, which was not found in Americans (Ishii et al., 2014). These findings suggest that SS carriers are more sensitive to environmental changes, but only in the presence of environmental changes of cultural importance.

There are a number of important limitations in the present study in need to be addressed. First, the present sample investigated autistic traits in subclinical populations and did not focus on patients with an autism diagnosis (or IUD). Future studies should also take into account patient samples, and these should be best also characterized via structural interviews. Second, it should be recognized that the effect of one single polymorphism on complex psychological/psychiatric phenotypes is small and results should not be over‐interpreted. In particular, the 5‐HTTLPR by adverse environment interactions on depression has been shown not to be robust (Culverhouse et al., 2018) and we also hint toward the many problems to be faced at the moment when running genetic association studies (for details see Montag et al., 2020). Beyond that criticism, the nature of both autistic and IUD conditions is complex, and a sole focus on serotonin—such as in the present work—naturally brings limitations. For instance, several studies investigating molecular genetics targets related to the dopamine, oxytocin, and cholinergic systems in the realm of IUD showed potential other candidates of relevance in the molecular study of IUD (Han et al., 2007; Kim et al., 2011; Montag et al., 2012; Sariyska et al., 2018; Sindermann et al., 2021). The same is true for the autistic traits where other molecules aside from serotonin are involved (Li et al., 2012; Ramaswami & Geschwind, 2018). Finally, we mention that epigenetics could be a logical target for future study, especially when studying the molecular aspects of human behavior in different cultures (for an exemplary SLC6A4 epigenetics paper investigating depression in a German sample, see the paper by Sanwald et al. (2021)). Of note, differences in psychological dimensions such as power distance or collectivism/individualism might be of further interest when studying the IUD‐complex in Germany and China (Montag et al., 2016)—also when being more interested in the molecular aspects of IUD tendencies.

### 4.1 Implications

As the genetic marker rs25531—modulating the effect of 5‐HTTLPR on the SLC6A4 gene's mRNA levels—to our knowledge has not been investigated so far in the 5‐HTTLPR–autism research, we examined the associations between genetic markers (5‐HTTLPR/rs25531) and individual differences in autistic traits in two samples with different ethnic background stemming from China and Germany. Beyond this, we also relate 5‐HTTLPR/rs25531 genetics not only to autistic traits in the present work, but also to IUD, as a growing number of studies have shown overlap between these constructs. Our data suggests that the low expressing allele S' (S, L_G_) appears to be a putative risk allele for autistic traits in both Chinese and German samples, in particular in the homozygous S'S genotype constellation. Furthermore, our analyses indicate that both 5‐HTTLPR/rs25531 and cultural differences might be of relevance to understand associations between autistic traits and IUD tendencies, but this needs to be further backed up in future studies. As one can see, the present study raises a few relevant issues to be considered in further molecular investigations of ASD research. Further studies are needed to corroborate the relevance of our results.

## CONFLICT OF INTEREST

The authors declare that they have no competing interests.

### PEER REVIEW

The peer review history for this article is available at: https://publons.com/publon/10.1002/brb3.2747.

## AUTHOR CONTRIBUTIONS

Christian Montag and YingYing Zhang designed the present study. YingYing Zhang and Helena Schmitt did the genetic analysis. Ying‐Ying Zhang conducted the statistical analysis and wrote the first version of the manuscript. Shuxia Yao checked the statistical analysis. Helena Schmitt double checked the statistical analysis. Shuxia Yao, Helena Schmitt, Christian Montag, Benjamin Becker, and Keith M. Kendrick critically revised the manuscript and approved the final version of the manuscript for submission.

## Supporting information

Supplementary Table 1. Statistical tests for normal distribution of age and all scales under investigation by Skewness and Kurtosis tests in the Chinese and German samples.
Supplementary Table 2. Partial Spearman rank correlations between AQ and s‐IAT scores in China and Germany and then spilt by 5‐HTTLPR/rs25531 genotypes, controlled for sex and age.
Supplementary Table 3. Zero‐order bivariate correlations between the s‐IAT scores and AQ total and subscale scores in the smaller Chinese and German samples.
Supplementary Table 4. The reliabilities (Cronbach's α) of variables under investigation in the smaller Chinese sample.
Supplementary Table 5. Demographic and questionnaire scores in the smaller Chinese sample according to sex and 5‐HTTLPR/rs25531 genotypes.
Supplementary Table 6. Partial correlations between AQ and s‐IAT scores in the smaller Chinese and German samples, controlled for sex and age.
Supplementary Table 7. Partial Spearman rank correlations in the smaller Chinese and German samples, controlled for sex and age.

## Data Availability

The datasets used and/or analyzed during the current study are available from the corresponding author on reasonable request.

## NOTE

1
https://www.ibm.com/support/pages/calculation-bonferroni-adjusted-p-values.

## References

[brb32747-bib-0001] Arieff, Z. , Kaur, M. , Gameeldien, H. , Van Der Merwe, L. , & Bajic, V. B. (2010). 5‐HTTLPR polymorphism: Analysis in South African autistic individuals. Human Biology, 82(3), 291–300. 20649385 10.3378/027.082.0303

[brb32747-bib-0002] Austin, E. J. (2005). Personality correlates of the broader autism phenotype as assessed by the Autism Spectrum Quotient (AQ). Personality and Individual Differences, 38(2), 451–460.

[brb32747-bib-0003] Baron‐Cohen, S. , Wheelwright, S. , Skinner, R. , Martin, J. , & Clubley, E. (2001). The autism‐spectrum quotient (AQ): Evidence from asperger syndrome/high‐functioning autism, males and females, scientists and mathematicians. Journal of Autism and Developmental Disorders, 31(1), 5–17. 11439754 10.1023/a:1005653411471

[brb32747-bib-0004] Bralten, J. , Van Hulzen, K. , Martens, M. , Galesloot, T. , Vasquez, A. A. , Kiemeney, L. , Buitelaar, J. K. , Muntjewerff, J. W. , Franke, B. , & Poelmans, G. (2018). Autism spectrum disorders and autistic traits share genetics and biology. Molecular Psychiatry, 23(5), 1205–1212. 28507316 10.1038/mp.2017.98 PMC5984081

[brb32747-bib-0005] Brand, M. , Young, K. S. , Laier, C. , Wölfling, K. , & Potenza, M. N. (2016). Integrating psychological and neurobiological considerations regarding the development and maintenance of specific Internet‐use disorders: An Interaction of Person‐Affect‐Cognition‐Execution (I‐PACE) model. Neuroscience & Biobehavioral Reviews, 71, 252–266. 27590829 10.1016/j.neubiorev.2016.08.033

[brb32747-bib-0006] Caspi, A. , Sugden, K. , Moffitt, T. E. , Taylor, A. , Craig, I. W. , Harrington, H. , McClay, J. , Mill, J. , Martin, J. , Poulton, R. , & Braithwaite, A. (2003). Influence of life stress on depression: Moderation by a polymorphism in the 5‐HTT gene. Science, 301(5631), 386–389. 12869766 10.1126/science.1083968

[brb32747-bib-0007] Cavalli‐Sforza, L. L. , & Feldman, M. W. (1981). Cultural transmission and evolution: A quantitative approach. Princeton University Press. 7300842

[brb32747-bib-0008] Cerniglia, L. , Cimino, S. , Marzilli, E. , Pascale, E. , & Tambelli, R. (2020). Associations among internet addiction, genetic polymorphisms, family functioning, and psychopathological risk: Cross‐sectional exploratory study. JMIR Mental Health, 7(12), e17341. 33361057 10.2196/17341 PMC7790611

[brb32747-bib-0009] Chiao, J. Y. , & Blizinsky, K. D. (2010). Culture–gene coevolution of individualism–collectivism and the serotonin transporter gene. Proceedings of the Royal Society B: Biological Sciences, 277(1681), 529–537. 10.1098/rspb.2009.1650 PMC2842692 19864286

[brb32747-bib-0010] Cho, I. H. , Yoo, H. J. , Park, M. , Lee, Y. S. , & Kim, S. A. (2007). Family‐based association study of 5‐HTTLPR and the 5‐HT2A receptor gene polymorphisms with autism spectrum disorder in Korean trios. Brain research, 1139, 34–41. 17280648 10.1016/j.brainres.2007.01.002

[brb32747-bib-0011] Culverhouse, R. C. , Saccone, N. L. , Horton, A. C. , Ma, Y. , Anstey, K. J. , Banaschewski, T. , Burmeister, M. , Cohen‐Woods, S. , Etain, B. , Fisher, H. L. , Goldman, N. , Guillaume, S. , Horwood, J. , Juhasz, G. , Lester, K. J. , Mandelli, L. , Middeldorp, C. M. , Olié, E. , Villafuerte, S. , …, Fisher, H. L. (2018). Collaborative meta‐analysis finds no evidence of a strong interaction between stress and 5‐HTTLPR genotype contributing to the development of depression. Molecular psychiatry, 23(1), 133–142. 28373689 10.1038/mp.2017.44 PMC5628077

[brb32747-bib-0012] Elhami Athar, M. , Ebrahimi, A. , Karimi, S. , Esmailzadeh, R. , Mousavi Asl, E. , Azizi, M. , Heidarzadeh, S. , Siahkamari, E. , Sharifi, A. , & Ramezani Farani, A. (2021). Comparison of autistic traits between iranian students with different ethnic backgrounds: A cross‐cultural study. Frontiers in Psychiatry, 12, 744180. 34955912 10.3389/fpsyt.2021.744180 PMC8695767

[brb32747-bib-0013] Ellis, P. D. (2010). The essential guide to effect sizes: Statistical power, meta‐analysis, and the interpretation of research results. Cambridge University Press.

[brb32747-bib-0014] Finkenauer, C. , Pollmann, M. M. , Begeer, S. , & Kerkhof, P. (2012). Brief report: Examining the link between autistic traits and compulsive Internet use in a non‐clinical sample. Journal of Autism and Developmental Disorders, 42(10), 2252–2256. 22350338 10.1007/s10803-012-1465-4

[brb32747-bib-0015] Folstein, S. , & Rutter, M. (1977). Infantile autism: A genetic study of 21 twin pairs. Journal of Child Psychology and Psychiatry, 18(4), 297–321. 562353 10.1111/j.1469-7610.1977.tb00443.x

[brb32747-bib-0016] Freeth, M. , Bullock, T. , & Milne, E. (2013). The distribution of and relationship between autistic traits and social anxiety in a UK student population. Autism, 17(5), 571–581. 22987896 10.1177/1362361312445511

[brb32747-bib-0017] Freeth, M. , Sheppard, E. , Ramachandran, R. , & Milne, E. (2013). A cross‐cultural comparison of autistic traits in the UK, India and Malaysia. Journal of Autism and Developmental Disorders, 43(11), 2569–2583. 23494561 10.1007/s10803-013-1808-9

[brb32747-bib-0018] Gressier, F. , Calati, R. , & Serretti, A. (2016). 5‐HTTLPR and gender differences in affective disorders: A systematic review. Journal of Affective Disorders, 190, 193–207. 26519640 10.1016/j.jad.2015.09.027

[brb32747-bib-0019] Han, D. H. , Lee, Y. S. , Yang, K. C. , Kim, E. Y. , Lyoo, I. K. , & Renshaw, P. F. (2007). Dopamine genes and reward dependence in adolescents with excessive internet video game play. Journal of addiction medicine, 1(3), 133–138. 21768948 10.1097/ADM.0b013e31811f465f

[brb32747-bib-0020] Heils, A. , Teufel, A. , Petri, S. , Stöber, G. , Riederer, P. , Bengel, D. , & Lesch, K. P. (1996). Allelic variation of human serotonin transporter gene expression. Journal of neurochemistry, 66(6), 2621–2624. 8632190 10.1046/j.1471-4159.1996.66062621.x

[brb32747-bib-0021] Ho, P.‐S. , Chen, C.‐L. , Chang, C.‐C. , Chang, H.‐A. , Yeh, Y.‐W. , Liang, C.‐S. , …, & Huang, C.‐C. (2016). The serotonin transporter gene (triallelic 5‐HTTLPR polymorphism) may associate with male depression in Han Chinese population. Journal of Medical Sciences, 36(2), 59.

[brb32747-bib-0022] Hu, X. , Lipsky, R. H. , Zhu, G. , Akhtar, L. A. , Taubman, J. , Greenberg, B. D. , Xu, K. , Arnold, P. D. , Richter, M. A. , Murphy, D. L. , Goldman, D. , & Kennedy, J. L. (2006). Serotonin transporter promoter gain‐of‐function genotypes are linked to obsessive‐compulsive disorder. American Journal of Human Genetics, 78(5), 815–826. 16642437 10.1086/503850 PMC1474042

[brb32747-bib-0023] Ishii, K. , Kim, H. S. , Sasaki, J. Y. , Shinada, M. , & Kusumi, I. (2014). Culture modulates sensitivity to the disappearance of facial expressions associated with serotonin transporter polymorphism (5‐HTTLPR). Culture and Brain, 2(1), 72–88.

[brb32747-bib-0024] Juhasz, G. , Gonda, X. , Hullam, G. , Eszlari, N. , Kovacs, D. , Lazary, J. , Pap, D. , Petschner, P. , Elliott, R. , Muir Anderson, I. , Antal, P. , Lesch, K.‐P. , Bagdy, G. , & Deakin, J. F. W. (2015). Variability in the effect of 5‐HTTLPR on depression in a large European population: The role of age, symptom profile, type and intensity of life stressors. PLoS One, 10(3), e0116316. 25747798 10.1371/journal.pone.0116316 PMC4351953

[brb32747-bib-0025] Kim, S. H. , Baik, S.‐H. , Park, C. S. , Kim, S. J. , Choi, S. W. , & Kim, S. E. (2011). Reduced striatal dopamine D2 receptors in people with Internet addiction. Neuroreport, 22(8), 407–411. 21499141 10.1097/WNR.0b013e328346e16e

[brb32747-bib-0026] Laplante, D. P. , Simcock, G. , Cao‐Lei, L. , Mouallem, M. , Elgbeili, G. , Brunet, A. , Cobham, V. , Kildea, S. , & King, S. (2019). The 5‐HTTLPR polymorphism of the serotonin transporter gene and child's sex moderate the relationship between disaster‐related prenatal maternal stress and autism spectrum disorder traits: The QF2011 Queensland flood study. Development and Psychopathology, 31(4), 1395–1409. 30394245 10.1017/S0954579418000871

[brb32747-bib-0027] Lee, Y. S. , Han, D. H. , Yang, K. C. , Daniels, M. A. , Na, C. , Kee, B. S. , & Renshaw, P. F. (2008). Depression like characteristics of 5HTTLPR polymorphism and temperament in excessive internet users. Journal of Affective Disorders, 109(1–2), 165–169. 18045695 10.1016/j.jad.2007.10.020

[brb32747-bib-0028] Lesch, K.‐P. , Bengel, D. , Heils, A. , Sabol, S. Z. , Greenberg, B. D. , Petri, S. , Benjamin, J. , Müller, C. R. , Hamer, D. H. , & Murphy, D. L. (1996). Association of anxiety‐related traits with a polymorphism in the serotonin transporter gene regulatory region. Science, 274(5292), 1527–1531. 8929413 10.1126/science.274.5292.1527

[brb32747-bib-0029] Lesch, K. , Meyer, J. , Glatz, K. , Flügge, G. , Hinney, A. , Hebebrand, J. , Klauck, S. M. , Poustka, A. , Poustka, F. , Mössner, R. , Riederer, P. , Heils, A. , & Bengel, D. (1997). The 5‐HT transporter gene‐linked polymorphic region (5‐HTTLPR) in evolutionary perspective: Alternative biallelic variation in rhesus monkeys. Journal of Neural Transmission, 104(11–12), 1259–1266. 9503271 10.1007/BF01294726

[brb32747-bib-0030] Li, X. , Zou, H. , & Brown, W. T. (2012). Genes associated with autism spectrum disorder. Brain Research Bulletin, 88(6), 543–552. 22688012 10.1016/j.brainresbull.2012.05.017

[brb32747-bib-0031] Little, K. Y. , McLaughlin, D. P. , Zhang, L. , Livermore, C. S. , Dalack, G. W. , McFinton, P. R. , DelProposto, Z. S. , Hill, E. , Cassin, B. J. , Cook, E. H. , & Watson, S. J. (1998). Cocaine, ethanol, and genotype effects on human midbrain serotonin transporter binding sites and mRNA levels. American Journal of Psychiatry, 155(2), 207–213. 9464199 10.1176/ajp.155.2.207

[brb32747-bib-0032] Lu, H. , Yu, J. , Wang, J. , Wu, L. , Xiao, H. , & Gao, R. (2016). Simultaneous quantification of neuroactive dopamine serotonin and kynurenine pathway metabolites in gender‐specific youth urine by ultra performance liquid chromatography tandem high resolution mass spectrometry. Journal of Pharmaceutical and Biomedical Analysis, 122, 42–51. 26845201 10.1016/j.jpba.2016.01.031

[brb32747-bib-0033] Lundström, S. , Chang, Z. , Råstam, M. , Gillberg, C. , Larsson, H. , Anckarsäter, H. , & Lichtenstein, P. (2012). Autism spectrum disorders and autisticlike traits: Similar etiology in the extreme end and the normal variation. Archives of General Psychiatry, 69(1), 46–52. 22213788 10.1001/archgenpsychiatry.2011.144

[brb32747-bib-0034] Meguid, N. A. , Gebril, O. H. , & Khalil, R. O. (2015). A study of blood serotonin and serotonin transporter promoter variant (5‐HTTLPR) polymorphism in Egyptian autistic children. Advanced Biomedical Research, 4. 26015920 10.4103/2277-9175.156658 PMC4434456

[brb32747-bib-0035] Montag, C. , & Becker, B. (2020). Internet and smartphone use disorder in Asia. 10.1016/j.addbeh.2020.106380 32220564

[brb32747-bib-0069] Montag, C. , Duke, E. , Sha, P. , Zhou, M. , Sindermann, C. , & Li, M. (2016). Does acceptance of power distance influence propensities for problematic Internet use? Evidence from a cross‐cultural study. Asia‐Pacific Psychiatry, 8(4), 296–301. 26676764 10.1111/appy.12229

[brb32747-bib-0036] Montag, C. , Ebstein, R. P. , Jawinski, P. , & Markett, S. (2020). Molecular genetics in psychology and personality neuroscience: On candidate genes, genome wide scans, and new research strategies. Neuroscience & Biobehavioral Reviews, 118, 163–174. 32681937 10.1016/j.neubiorev.2020.06.020

[brb32747-bib-0037] Montag, C. , Kirsch, P. , Sauer, C. , Markett, S. , & Reuter, M. (2012). The role of the CHRNA4 gene in Internet addiction: A case‐control study. Journal of Addiction Medicine, 6(3), 191–195. 22722381 10.1097/ADM.0b013e31825ba7e7

[brb32747-bib-0038] Montag, C. , & Reuter, M. (2017). Molecular genetics, personality, and internet addiction revisited. In Internet Addiction (pp. 141–160). Springer.

[brb32747-bib-0039] Montag, C. , Wegmann, E. , Sariyska, R. , Demetrovics, Z. , & Brand, M. (2021). How to overcome taxonomical problems in the study of Internet use disorders and what to do with “smartphone addiction”? Journal of behavioral addictions, 9(4), 908–914. 31668089 10.1556/2006.8.2019.59 PMC8969715

[brb32747-bib-0040] Nakamura, M. , Ueno, S. , Sano, A. , & Tanabe, H. (2000). The human serotonin transporter gene linked polymorphism (5‐HTTLPR) shows ten novel allelic variants. Molecular Psychiatry, 5(1), 32–38. 10673766 10.1038/sj.mp.4000698

[brb32747-bib-0041] Nishizawa, S. , Benkelfat, C. , Young, S. , Leyton, M. , Mzengeza, S. D. , De Montigny, C. , Blier, P. , & Diksic, M. (1997). Differences between males and females in rates of serotonin synthesis in human brain. Proceedings of the National Academy of Sciences, 94(10), 5308–5313. 10.1073/pnas.94.10.5308 PMC24674 9144233

[brb32747-bib-0042] Nonweiler, J. , Rattray, F. , Baulcomb, J. , Happé, F. , & Absoud, M. (2020). Prevalence and associated factors of emotional and behavioural difficulties during COVID‐19 pandemic in children with neurodevelopmental disorders. Children, 7(9), 128. 10.3390/children7090128 PMC7552706 32899799

[brb32747-bib-0043] Nuñez‐Rios, D. , Chaskel, R. , Lopez, A. , Galeano, L. , & Lattig, M. (2020). The role of 5‐HTTLPR in autism spectrum disorder: New evidence and a meta‐analysis of this polymorphism in Latin American population with psychiatric disorders. PLoS One, 15(7), e0235512. 10.1371/journal.pone.0235512 PMC7332001 32614901

[brb32747-bib-0044] Paik, S.‐H. , Choi, M. R. , Kwak, S. M. , Bang, S. H. , Chun, J.‐W. , Kim, J.‐Y. , Choi, J. , Cho, H. , Jeong, J.‐E. , & Kim, D.‐J. (2017). An association study of Taq1A ANKK1 and C957T and− 141C DRD2 polymorphisms in adults with internet gaming disorder: A pilot study. Annals of General Psychiatry, 16(1), 1–8. 29234453 10.1186/s12991-017-0168-9 PMC5721653

[brb32747-bib-0045] Pawlikowski, M. , Altstötter‐Gleich, C. , & Brand, M. (2013). Validation and psychometric properties of a short version of Young's Internet Addiction Test. Computers in Human Behavior, 29(3), 1212–1223.

[brb32747-bib-0046] Ramaswami, G. , & Geschwind, D. H. (2018). Genetics of autism spectrum disorder. Handbook of Clinical Neurology, 147, 321–329. 29325621 10.1016/B978-0-444-63233-3.00021-X

[brb32747-bib-0047] Richerson, P. J. , Boyd, R. , & Henrich, J. (2010). Gene‐culture coevolution in the age of genomics. Proceedings of the National Academy of Sciences, 107(Supplement2), 8985–8992. 10.1073/pnas.0914631107 PMC3024025 20445092

[brb32747-bib-0048] Romano, M. , Truzoli, R. , Osborne, L. A. , & Reed, P. (2014). The relationship between autism quotient, anxiety, and internet addiction. Research in Autism Spectrum Disorders, 8(11), 1521–1526.

[brb32747-bib-0049] Ronald, A. , Happe, F. , Price, T. S. , Baroncohen, S. , & Plomin, R. (2006). Phenotypic and genetic overlap between autistic traits at the extremes of the general population. Journal of the American Academy of Child and Adolescent Psychiatry, 45(10), 1206–1214. 17003666 10.1097/01.chi.0000230165.54117.41

[brb32747-bib-0050] Ruzich, E. , Allison, C. , Smith, P. , Watson, P. , Auyeung, B. , Ring, H. , & Baron‐Cohen, S. (2015). Measuring autistic traits in the general population: A systematic review of the Autism‐Spectrum Quotient (AQ) in a nonclinical population sample of 6,900 typical adult males and females. Molecular Autism, 6(1), 1–12. 25874074 10.1186/2040-2392-6-2 PMC4396128

[brb32747-bib-0051] Sakai, Y. , Nishikawa, M. , Leyton, M. , Benkelfat, C. , Young, S. N. , & Diksic, M. (2006). Cortical trapping of α‐[11C] methyl‐l‐tryptophan, an index of serotonin synthesis, is lower in females than males. Neuroimage, 33(3), 815–824. 16996279 10.1016/j.neuroimage.2006.08.004

[brb32747-bib-0052] Sanwald, S. , Widenhorn‐Müller, K. , Schönfeldt‐Lecuona, C. , Montag, C. , & Kiefer, M. (2021). Factors related to age at depression onset: The role of SLC6A4 methylation, sex, exposure to stressful life events and personality in a sample of inpatients suffering from major depression. Bmc Psychiatry [Electronic Resource], 21(1), 1–14. 10.1186/s12888-021-03166-6 PMC7995700 33765975

[brb32747-bib-0053] Sariyska, R. , Rathner, E.‐M. , Baumeister, H. , & Montag, C. (2018). Feasibility of linking molecular genetic markers to real‐world social network size tracked on smartphones. Frontiers in Neuroscience, 12, 945. 30618574 10.3389/fnins.2018.00945 PMC6305317

[brb32747-bib-0054] Sindermann, C. , Sariyska, R. , Elhai, J. D. , & Montag, C. (2021). Molecular genetics of neurotransmitters and neuropeptides involved in Internet use disorders including first insights on a potential role of hypothalamus’ oxytocin hormone. Handbook of Clinical Neurology, 182, 389–400. 34266607 10.1016/B978-0-12-819973-2.00026-5

[brb32747-bib-0055] Spada, M. M. (2014). An overview of problematic Internet use. Addictive Behaviors, 39(1), 3–6. 24126206 10.1016/j.addbeh.2013.09.007

[brb32747-bib-0055a] Stodt, B. , Brand, M. , Sindermann, C. , Wegmann, E. , Li, M. , Zhou, M. , … & Montag, C. (2018). Investigating the effect of personality, internet literacy, and use expectancies in internet‐use disorder: A comparative study between China and Germany. International journal of environmental research and public health, 15(4), 579. 10.3390/ijerph15040579 PMC5923621 29570663

[brb32747-bib-0056] Sun, C. , Spathis, R. , Lum, J. K. , Sankaranarayanan, K. , & Chan, C. W. (2016). Genetic‐linked Inattentiveness Protects Individuals from Internet Overuse: A Genetic Study of Internet Overuse Evaluating Hypotheses Based on Addiction, Inattention, Novelty‐seeking and Harm‐avoidance. Informing Science, 19.

[brb32747-bib-0057] Tabachnick, B. G. , & Fidell, L. S. (1996). *Using multivariate statistics* . Harper Collins.

[brb32747-bib-0058] Tick, B. , Bolton, P. , Happé, F. , Rutter, M. , & Rijsdijk, F. (2016). Heritability of autism spectrum disorders: A meta‐analysis of twin studies. Journal of Child Psychology and Psychiatry, 57(5), 585–595. 26709141 10.1111/jcpp.12499 PMC4996332

[brb32747-bib-0059] Tordjman, S. , Gutknecht, L. , Carlier, M. , Spitz, E. , Antoine, C. , Slama, F. , Carsalade, V. , Cohen, D. J. , Ferrari, P. , Anderson, G. M. , & Roubertoux, P. (2001). Role of the serotonin transporter gene in the behavioral expression of autism. Molecular Psychiatry, 6(4), 434–439. 11443529 10.1038/sj.mp.4000873

[brb32747-bib-0060] Wakabayashi, A. , Baron‐Cohen, S. , Wheelwright, S. , & Tojo, Y. (2006). The Autism‐Spectrum Quotient (AQ) in Japan: A cross‐cultural comparison. Journal of Autism and Developmental Disorders, 36(2), 263–270. 16586157 10.1007/s10803-005-0061-2

[brb32747-bib-0061] Wang, H. , Yin, F. , Gao, J. , & Fan, X. (2019). Association between 5‐HTTLPR polymorphism and the risk of autism: A meta‐analysis based on case‐control studies. Frontiers in Psychiatry, 10, 51. 30814960 10.3389/fpsyt.2019.00051 PMC6381045

[brb32747-bib-0062] Wei, H. , Zhu, Y. , Wang, T. , Zhang, X. , Zhang, K. , & Zhang, Z. (2021). Genetic risk factors for autism‐spectrum disorders: A systematic review based on systematic reviews and meta‐analysis. Journal of Neural Transmission, 1–18. 10.1007/s00702-021-02360-w 34115189

[brb32747-bib-0063] Wendland, J. , Martin, B. , Kruse, M. , Lesch, K. , & Murphy, D. (2006). Simultaneous genotyping of four functional loci of human SLC6A4, with a reappraisal of 5‐HTTLPR and rs25531. Molecular Psychiatry, 11(3), 224–226. 16402131 10.1038/sj.mp.4001789

[brb32747-bib-0064] Woodbury‐Smith, M. R. , Robinson, J. , Wheelwright, S. , & Baron‐Cohen, S. (2005). Screening adults for Asperger syndrome using the AQ: A preliminary study of its diagnostic validity in clinical practice. Journal of Autism and Developmental Disorders, 35(3), 331–335. 16119474 10.1007/s10803-005-3300-7

[brb32747-bib-0065] Young, K. S. (1998). Caught in the net: How to recognize the signs of internet addiction—and a winning strategy for recovery. John Wiley & Sons.

[brb32747-bib-0066] Zalsman, G. , Huang, Y.‐y. , Oquendo, M. A. , Burke, A. K. , Hu, X.‐Z. , Brent, D. A. , Ellis, S. P. , Goldman, D. , & Mann, J. J. (2006). Association of a triallelic serotonin transporter gene promoter region (5‐HTTLPR) polymorphism with stressful life events and severity of depression. American Journal of Psychiatry, 163(9), 1588–1593. 16946185 10.1176/ajp.2006.163.9.1588

[brb32747-bib-0067] Zhang, Y. , Sindermann, C. , Kendrick, K. M. , Becker, B. , & Montag, C. (2021). Individual differences in tendencies toward internet use disorder, internet literacy and their link to autistic traits in both China and Germany. Frontiers in Psychiatry, 12. 10.3389/fpsyt.2021.638655 PMC8502933 34646170

[brb32747-bib-0068] Zhao, X. , Li, X. , Song, Y. , & Shi, W. (2019). Autistic traits and prosocial behaviour in the general population: Test of the mediating effects of trait empathy and state empathic concern. Journal of Autism and Developmental Disorders, 49(10), 3925–3938. 30203310 10.1007/s10803-018-3745-0

