# Supplementary material for: Molecular genetic associations between a prominent serotonin transporter gene polymorphism (5‐HTTLPR/rs25531) and individual differences in tendencies toward autistic traits and generalized internet use disorder in China and Germany
Source: Brain Behav. 2022 Sep 15;12(10):e2747. doi: 10.1002/brb3.2747 (PMC9575603; doi:10.1002/brb3.2747)
Supplement: Supplementary file 1 — Supplementary Table 1. Statistical tests for normal distribution of age and all scales under investigation by Skewness and Kurtosis tests in the Chinese and German samples. Supplementary Table 2. Partial Spearman rank correlations between AQ and s‐IAT scores in China and Germany and then spilt by 5‐HTTLPR/rs25531 genotypes, controlled for sex and age. Supplementary Table 3. Zero‐order bivariate correlations between the s‐IAT scores and AQ total and subscale scores in the smaller Chinese and German samples. Supplementary Table 4. The reliabilities (Cronbach's α) of variables under investigation in the smaller Chinese sample. Supplementary Table 5. Demographic and questionnaire scores in the smaller Chinese sample according to sex and 5‐HTTLPR/rs25531 genotypes. Supplementary Table 6. Partial correlations between AQ and s‐IAT scores in the smaller Chinese and German samples, controlled for sex and age. Supplementary Table 7. Partial Spearman rank correlations in the smaller Chinese and German samples, controlled for sex and age. [file BRB3-12-e2747-s001.docx]

**Molecular genetic associations between a prominent serotonin transporter gene polymorphism (5-HTTLPR/rs25531) and individual differences in tendencies towards autistic traits and generalized Internet Use Disorder in China and Germany**

YingYing Zhang^a*^, Shuxia Yao^b^, Helena Schmitt^a^, Benjamin Becker^b^, Keith M. Kendrick^b^ & Christian Montag^a, b*^

^a^Department of Molecular Psychology, Institute of Psychology and Education, Ulm University, Ulm, Germany

^b^The Clinical Hospital of Chengdu Brain Science Institute, Ministry of Education Key Lab for Neuroinformation, University of Electronic Science and Technology of China, Chengdu, China

**Part 1**

**1. Distributions of scales under investigation presented separately for the Chinese and German samples**

Skewness and kurtosis were computed for scales under investigation. In particular, normality assumptions were considered met if the z-scores for skewness and kurtosis ranged from -2 to +2 ([Curran et al., 1996](#_ENREF_1)). Results of the skewness and kurtosis are depicted in detail in **Supplementary Table 1** (separately for China and Germany).

**Supplementary Table 1.**

Statistical tests for normal distribution of age and all scales under investigation by Skewness and Kurtosis tests in the Chinese and German samples.

|  | **Chinese sample**  (n = 540) | | | | **German sample**  (n = 563) | | | |
| --- | --- | --- | --- | --- | --- | --- | --- | --- |
|  | **Skewness** | | **Kurtosis** | | **Skewness** | | **Kurtosis** | |
|  | Statistic | SE | Statistic | SE | Statistic | SE | Statistic | SE |
| **AQ total** | -0.10 | 0.11 | -0.24 | 0.21 | 0.70 | 0.10 | 1.09 | 0.21 |
| small AQ | 0.00 | 0.11 | -0.32 | 0.21 | 0.72 | 0.10 | 0.66 | 0.21 |
| AQ SS | 0.16 | 0.11 | -0.91 | 0.21 | 1.07 | 0.10 | 0.62 | 0.21 |
| AQ DP | -0.20 | 0.11 | -0.32 | 0.21 | -0.15 | 0.10 | -0.70 | 0.21 |
| AQ CM | 0.40 | 0.11 | -0.67 | 0.21 | 1.36 | 0.10 | 1.82 | 0.21 |
| **s-IAT total** | 0.48 | 0.11 | 0.48 | 0.21 | 1.77 | 0.10 | 3.90 | 0.21 |
| s-IAT LoC/TM | 0.24 | 0.11 | -0.09 | 0.21 | 1.20 | 0.10 | 1.60 | 0.21 |
| s-IAT C/SP | 0.70 | 0.11 | 0.84 | 0.21 | 3.38 | 0.10 | 13.35 | 0.21 |

Abbreviations: AQ, the Adult Autism Spectrum Quotient; SS, *social skills*; DP, *details/pattern*; CM, *communication/mindreading*; s-IAT, the short Internet Addiction Test; LoC/TM, *loss of control/time management*; C/SP, *craving/social problems.*

**2. Associations between age and the variables of interest**

For correlations between age and AQ total scores and AQ subscales’ scores, significant correlations were only observed in the German sample (AQ total scores, r = 0.09, *p =* 0.043*;* AQ *social skills* subscale, r = 0.10, *p =* 0.022). For correlations between age and s-IAT scores and s-IAT subscales’ scores, significantly negative correlations between age and s-IAT total scores (China: rs = - 0.12, *p* = 0.007; Germany: rs = - 0.10, *p* = 0.019), as well as the s-IAT *loss of control/time management* subscale (China: rs = - 0.13, *p* = 0.002; Germany: rs = - 0.13, *p =* 0.002) were found in both samples. Besides, age was significantly correlated with s-IAT *craving/social problems* (rs = - 0.09, *p* = 0.046) in the Chinese sample.

**3. Associations between autistic traits and IUD tendencies with non-parametric partial correlations**

**Supplementary Table 2.**

Partial Spearman rank correlations between AQ and s-IAT scores in China and Germany and then spilt by 5-HTTLPR/rs25531 genotypes, controlled for sex and age.

| **Variables** | **Chinese sample**  (n = 540) | | |  | **German sample**  (n = 563) | | |
| --- | --- | --- | --- | --- | --- | --- | --- |
| **Total sample** | s-IAT  total | s-IAT LoC/TM | s-IAT  C/SP |  | s-IAT  total | s-IAT LoC/TM | s-IAT  C/SP |
| AQ total | 0.18^***^ | 0.13^**^ | 0.21^***^ |  | 0.16^***^ | 0.12^**^ | 0.24^***^ |
| small AQ | 0.13^**^ | 0.08 | 0.16^***^ |  | 0.19^***^ | 0.15^***^ | 0.23^***^ |
| AQ SS | 0.10^*^ | 0.06 | 0.13^**^ |  | 0.15^***^ | 0.11^**^ | 0.24^***^ |
| AQ DP | -0.09^*^ | -0.08^*^ | -0.08 |  | 0.00 | -0.01 | 0.01 |
| AQ CM | 0.23^***^ | 0.18^***^ | 0.25^***^ |  | 0.24^***^ | 0.22^***^ | 0.24^***^ |
| **S’S’** | (n = 357) | | |  | (n = 118) | | |
| AQ total | 0.19^***^ | 0.16^**^ | 0.20^***^ |  | 0.00 | -0.06 | 0.17 |
| small AQ | 0.14^**^ | 0.10 | 0.17^**^ |  | 0.05 | 0.01 | 0.16 |
| AQ SS | 0.12^*^ | 0.10 | 0.14^**^ |  | 0.06 | 0.01 | 0.15 |
| AQ DP | -0.13^*^ | -0.13^*^ | -0.11^*^ |  | -0.15 | -0.18 | 0.00 |
| AQ CM | 0.26^***^ | 0.22^***^ | 0.27^***^ |  | 0.25^**^ | 0.22^*^ | 0.30^**^ |
| **S’L’** | (n = 166) | | |  | (n = 281) | | |
| AQ total | 0.24^**^ | 0.14 | 0.31^*^^**^ |  | 0.24^***^ | 0.21^***^ | 0.25^***^ |
| small AQ | 0.18^*^ | 0.10 | 0.24^**^ |  | 0.27^***^ | 0.24^***^ | 0.26^***^ |
| AQ SS | 0.13 | 0.06 | 0.18^*^ |  | 0.25^***^ | 0.23^***^ | 0.28^***^ |
| AQ DP | -0.02 | -0.01 | -0.04 |  | 0.07 | 0.07 | 0.02 |
| AQ CM | 0.19^*^ | 0.12 | 0.24^**^ |  | 0.24^***^ | 0.22^***^ | 0.23^***^ |
| **L’L’** | (n = 17) | | |  | (n = 164) | | |
| AQ total | -0.23 | -0.10 | -0.14 |  | 0.13 | 0.07 | 0.27^***^ |
| small AQ | -0.31 | -0.08 | -0.30 |  | 0.11 | 0.06 | 0.22^**^ |
| AQ SS | -0.58^*^ | -0.49 | -0.41 |  | 0.04 | -0.02 | 0.22^**^ |
| AQ DP | 0.31 | 0.47 | 0.10 |  | -0.01 | -0.03 | -0.02 |
| AQ CM | 0.10 | 0.36 | 0.07 |  | 0.22^**^ | 0.21^**^ | 0.20^*^ |

Abbreviations: AQ, the Adult Autism Spectrum Quotient; SS, *social skills*; DP, *details/pattern*; CM, *communication/mindreading*; s-IAT, the short Internet Addiction Test; LoC/TM, *loss of control/time management*; C/SP, *craving/social problems.* S’S’ (low expressing allele homozygotes): Sa/Sa, Sa/Lg, Lg/Lg; S’L’ (intermediate expressing allele heterozygotes): La/Sa, La/Lg, XL/Sa, XL/Lg, XXL/Sa, XXL/Lg; L’L’ (high expressing allele homozygotes): La/La, XL/La, XL/XL. **p* < 0.05, ***p* < 0.01, ****p* < 0.001.

**Part 2**

**1. Associations between autistic traits and IUD tendencies (on original five subscales level)**

To revisit the finding from Zhang et al. (2021) with our present dataset, we also calculated zero-order correlations between AQ score and s-IAT scores with exactly same AQ facets (original five subscales level; deleting the samples overlapping with [Zhang et al. (2021](#_ENREF_2)), this sample is named “smaller Chinese sample” in the following). Correlation patterns were similar in the investigated two samples (see **Supplementary Table 3**). In details, AQ facets including AQ *social skills* and AQ *communication* were significantly positively correlated with s-IAT total scores and s-IAT subscales’ scores in both samples. AQ’s *attention switching* facet was also positively correlated with the s-IAT total scores, although not reaching significance in the Chinese sample. The AQ *attention to details* facet was negatively correlated with s-IAT total scores in both samples, but this was only significant in the German sample. Moreover, the observed correlations across each AQ facet with the s-IAT were in the same direction as the reported results from Zhang et al. (2021) (for details see the Supplementary Table 5 in [Zhang et al. (2021](#_ENREF_2))), but see that significance levels reached were a bit different between these two studies.

**Supplementary Table 3.**

Zero-order bivariate correlations between the s-IAT scores and AQ total and subscale scores in the smaller Chinese and German samples.

|  | **Chinese sample**  (n = 374) | | |  | **German sample**  (n = 563) | | |
| --- | --- | --- | --- | --- | --- | --- | --- |
| **Variables** | s-IAT  total | s-IAT LoC/TM | s-IAT  C/SP |  | s-IAT  total | s-IAT LoC/TM | s-IAT  C/SP |
| AQ total | 0.11^*^ | 0.07 | 0.13^*^ |  | 0.21^***^ | 0.13^**^ | 0.29^***^ |
| AQ SS | 0.12^*^ | 0.11^*^ | 0.12^*^ |  | 0.27^***^ | 0.19^***^ | 0.34^***^ |
| AQ AS | 0.07 | 0.07 | 0.05 |  | 0.16^***^ | 0.12^**^ | 0.18^***^ |
| AQ AD | -0.07 | -0.08 | -0.05 |  | -0.11^**^ | -0.11^**^ | -0.08^*^ |
| AQ CO | 0.16^**^ | 0.11^*^ | 0.18^***^ |  | 0.29^***^ | 0.21^***^ | 0.33^***^ |
| AQ IM | 0.00 | -0.03 | 0.03 |  | 0.08 | 0.01 | 0.17^***^ |

Abbreviations: AQ, the Adult Autism Spectrum Quotient; SS, *social skills*; AS, *attention switching*; AD, *attention to details*; CO, *communication*; IM, imagination s-IAT, the short Internet Addiction Test; LoC/TM, *loss of control/time management*; C/SP, *carving/social problems.* **p* < 0.05, ***p* < 0.01, ****p* < 0.001.

**2. Results (excluding the overlapping participants with our earlier work in Zhang et al. (2021))**

Furthermore, we also ran the statistical analyses as in the manuscript without the overlapping participants regarding our earlier work ([Zhang et al., 2021](#_ENREF_2)). Results from the smaller Chinese sample are presented in the following tables (from Supplementary Table 4 to Supplementary Table 7), which were comparable to the results in the whole Chinese sample as presented in the main manuscript.

**Supplementary Table 4.**

The reliabilities (Cronbach’s α) of variables under investigation in the smaller Chinese sample.

| **Domain/variable** | **Chinese sample**  (n = 374) |
| --- | --- |
| **AQ scores** | **0.673** |
| AQ SS | 0.706 |
| AQ AS | 0.269 |
| AQ AD | 0.504 |
| AQ CO | 0.588 |
| AQ IM | 0.411 |
| **Small AQ scores** | **0.552** |
| AQ SS | 0.682 |
| AQ DP | 0.512 |
| AQ CM | 0.530 |
| **s-IAT** | **0.880** |
| s-IAT LoC/TM | 0.828 |
| s-IAT C/SP | 0.771 |

Abbreviations: AQ, the Adult Autism Spectrum Quotient; SS, *social skills*; AS, *attention switching;* AD, *attention to details*; CO, *communication*; IM, *imagination*; DP, *details/pattern*; CM, *communication/mindreading*; s-IAT, the short Internet Addiction Test; LoC/TM, *loss of control/time management*; C/SP, *craving/social problems.*

**Supplementary Table 5.**

Demographic and questionnaire scores in the smaller Chinese sample according to sex and 5-HTTLPR/rs25531 genotypes.

| **Variables** | **Total**  **sample** | **Sex** | | | **5-HTTLPR/rs25531 genotype** | | | |
| --- | --- | --- | --- | --- | --- | --- | --- | --- |
|  |  | **Males** | **Females** | ***p*** | **S’S’** | **S’L’** | **L’L’** | ***p*** |
| **Chinese**  **sample** | (n = 374) | (n = 237) | (n = 137) |  | (n = 247) | (n = 114) | (n = 13) |  |
| Age | 21.78 (2.22) | 21.85 (2.25) | 21.66 (2.17) | *0.431* | 21.80 (2.19) | 21.72 (2.27) | 22.08 (2.50) | *0.848* |
| AQ total | 21.93 (5.75) | 22.10 (5.81) | 21.63 (5.66) | *0.448* | 22.56 (5.77) | 20.75 (5.52) | 20.23 (5.97) | ***0.011*** |
| small AQ | 11.60 (3.57) | 11.81 (3.56) | 11.24 (3.56) | *0.134* | 12.13 (3.49) | 10.66 (3.52) | 10.00 (3.46) | ***< 0.001*** |
| AQ SS | 4.99 (2.73) | 5.00 (2.75) | 4.96 (2.71) | *0.870* | 5.28 (2.66) | 4.43 (2.77) | 4.31 (3.20) | ***0.015*** |
| AQ DP | 4.52 (1.82) | 4.76 (1.84) | 4.10 (1.71) | ***< 0.001*** | 4.63 (1.79) | 4.33 (1.86) | 4.08 (2.02) | *0.236* |
| AQ CM | 2.10 (1.54) | 2.05 (1.55) | 2.18 (1.53) | *0.412* | 2.21 (1.51) | 1.89 (1.60) | 1.62 (1.39) | *0.097* |
| s-IAT total | 32.60 (8.31) | 32.57 (8.59) | 32.64 (7.83) | *0.917* | 32.55 (8.21) | 32.63 (8.43) | 33.15 (9.81) | *0.833* |
| s-IAT LoC/TM | 18.04 (4.64) | 17.76 (4.67) | 18.53 (4.55) | *0.163* | 18.02 (4.66) | 18.11 (4.62) | 17.85 (4.58) | *0.851* |
| s-IAT C/SP | 14.56 (4.32) | 14.81 (4.56) | 14.11 (3.85) | 0.191 | 14.53 (4.23) | 14.52 (4.39) | 15.31 (5.54) | *0.824* |

Abbreviations: AQ, the Adult Autism Spectrum Quotient; SS, *social skills*; DP, *details/pattern*; CM, *communication/mindreading*; s-IAT, the short Internet Addiction Test; LoC/TM, *loss of control/time management*; C/SP, *craving/social problems.* S’S’ (low expressing allele homozygosity): S/S, S/L_G_ L_G_/L_G_; S’L’ (intermediate expressing allele heterozygosity): L_A_/S, L_A_/L_G_ XL/S, XL/L_G_, XXL/S, XXL/L_G_; L’L’ (high expressing allele homozygosity): L_A_/L_A,_ XL/L_A,_ XL/XL.

**Supplementary Table 6.**

Partial correlations between AQ and s-IAT scores in the smaller Chinese and German samples, controlled for sex and age.

|  | **Chinese sample**  (n = 374) | | |  | **German sample**  (n = 563) | | |  | **Fisher’s Z** | | |
| --- | --- | --- | --- | --- | --- | --- | --- | --- | --- | --- | --- |
| **Variables** | s-IAT  total | s-IAT LoC/TM | s-IAT  C/SP |  | s-IAT  total | s-IAT LoC/TM | s-IAT  C/SP |  | s-IAT  total | s-IAT LoC/TM | s-IAT  C/SP |
| AQ total | 0.11^*^ | 0.07 | 0.13^*^ |  | 0.20^***^ | 0.13^**^ | 0.27^***^ |  | -1.46 | -0.87 | -2.24^*^ |
| small AQ | 0.04 | 0.00 | 0.08 |  | 0.22^***^ | 0.16^***^ | 0.27^***^ |  | -2.74^**^ | -2.37^*^ | -2.91^**^ |
| AQ SS | 0.06 | 0.03 | 0.08 |  | 0.20^***^ | 0.14^***^ | 0.26^***^ |  | -2.18^*^ | -1.66 | -2.67^**^ |
| AQ DP | -0.14^**^ | -0.14^**^ | -0.12^*^ |  | -0.02 | -0.02 | -0.03 |  | -1.74 | -1.86 | -1.36 |
| AQ CM | 0.16^**^ | 0.12^*^ | 0.17^**^ |  | 0.32^***^ | 0.25^***^ | 0.35^***^ |  | -2.61^**^ | -1.99^*^ | -2.94^**^ |

Abbreviations: AQ, the Adult Autism Spectrum Quotient; SS, *social skills*; DP, *details/pattern*; CM, *communication/mindreading*; s-IAT, the short Internet Addiction Test; LoC/TM, *loss of control/time management*; C/SP, *craving/social problems.* **p* < 0.05, ***p* < 0.01, ****p* < 0.001.

**Supplementary Table 7.**

Partial Spearman rank correlations in the smaller Chinese and German samples, controlled for sex and age.

|  | **Chinese sample**  (n = 374) | | |  | **German sample**  (n = 563) | | |  | **Fisher’s Z** | | |
| --- | --- | --- | --- | --- | --- | --- | --- | --- | --- | --- | --- |
| **Variables** | s-IAT  total | s-IAT LoC/TM | s-IAT  C/SP |  | s-IAT  total | s-IAT LoC/TM | s-IAT  C/SP |  | s-IAT  total | s-IAT LoC/TM | s-IAT  C/SP |
| AQ total | 0.14^**^ | 0.10 | 0.17^***^ |  | 0.16^***^ | 0.12^**^ | 0.24^***^ |  | -0.35 | -0.31 | -1.12 |
| small AQ | 0.08 | 0.04 | 0.12^*^ |  | 0.19^***^ | 0.15^***^ | 0.23^***^ |  | -1.58 | -1.70 | -1.78 |
| AQ SS | 0.09 | 0.05 | 0.12^*^ |  | 0.15^***^ | 0.11^**^ | 0.24^***^ |  | -0.94 | -0.88 | -1.82 |
| AQ DP | -0.13^**^ | -0.13^*^ | -0.11^*^ |  | 0.00 | -0.01 | 0.01 |  | -1.93 | -1.83 | -1.78 |
| AQ CM | 0.19^***^ | 0.15^**^ | 0.20^***^ |  | 0.24^***^ | 0.22^***^ | 0.24^***^ |  | -0.91 | -1.14 | -0.65 |

Abbreviations: AQ, the Adult Autism Spectrum Quotient; SS, *social skills*; DP, *details/pattern*; CM, *communication/mindreading*; s-IAT, the short Internet Addiction Test; LoC/TM, *loss of control/time management*; C/SP, *craving/social problems.* **p* < 0.05, ***p* < 0.01, ****p* < 0.001.

**References**

Curran, P. J., West, S. G., & Finch, J. F. (1996). The robustness of test statistics to nonnormality and specification error in confirmatory factor analysis. *Psychological methods*, *1*(1), 16.

Zhang, Y., Sindermann, C., Kendrick, K. M., Becker, B., & Montag, C. (2021). Individual Differences in Tendencies Toward Internet Use Disorder, Internet Literacy and Their Link to Autistic Traits in Both China and Germany. *Frontiers in Psychiatry*, *12*.
